# Signaling through the *Salmonella* PbgA-LapB regulatory complex activates LpxC proteolysis and limits lipopolysaccharide biogenesis during stationary-phase growth

**DOI:** 10.1128/jb.00308-23

**Published:** 2024-03-27

**Authors:** Joshua A. Mettlach, Melina B. Cian, Medha Chakraborty, Zachary D. Dalebroux

**Affiliations:** 1 Department of Microbiology and Immunology, University of Oklahoma Health Sciences Center, Oklahoma City, Oklahoma, USA; Geisel School of Medicine at Dartmouth, Hanover, New Hampshire, USA

**Keywords:** LapB, YciM, PbgA, YejM, LapC, LpxC, lipopolysaccharide, LPS, lipid A, endotoxin, lipid A-core, signal transduction, stress response, cell envelope, outer membrane, Gram-negative

## Abstract

*Salmonella enterica* serovar Typhimurium (*S*. Typhimurium) controls lipopolysaccharide (LPS) biosynthesis by regulating proteolysis of LpxC, the rate-limiting enzyme and target of preclinical antibiotics. PbgA/YejM/LapC regulates LpxC levels and controls outer membrane (OM) LPS composition at the log-to-stationary phase transition. Suppressor substitutions in LPS assembly protein B (LapB/YciM) rescue the LPS and OM integrity defects of *pbgA*-mutant *S*. Typhimurium. We hypothesized that PbgA regulates LpxC proteolysis by controlling LapB’s ability to bind LpxC as a function of the growth phase. According to existing models, when nutrients are abundant, PbgA binds and restricts LapB from interacting with LpxC and FtsH, which limits LpxC proteolysis. However, when nutrients are limited, there is debate whether LapB dissociates from PbgA to bind LpxC and FtsH to enhance degradation. We sought to examine these models and investigate how the structure of LapB enables salmonellae to control LpxC proteolysis and LPS biosynthesis. Salmonellae increase LapB levels during the stationary phase to promote LpxC degradation, which limits lipid A-core production and increases their survival. The deletion of *lapB*, resulting in unregulated lipid A-core production and LpxC overabundance, leads to bacterial growth retardation. Tetratricopeptide repeats near the cytosol-inner membrane interface are sufficient for LapB to bind LpxC, and remarkably, LapB and PbgA interact in both growth phases, yet LpxC only associates with LapB in the stationary phase. Our findings support that PbgA-LapB exists as a constitutive complex in *S*. Typhimurium, which differentially binds LpxC to control LpxC proteolysis and limit lipid A-core biosynthesis in response to changes in the environment.

**IMPORTANCE:**

Antimicrobial resistance has been a costly setback for human health and agriculture. Continued pursuit of new antibiotics and targets is imperative, and an improved understanding of existing ones is necessary. LpxC is an essential target of preclinical trial antibiotics that can eliminate multidrug-resistant Gram-negative bacterial infections. LapB is a natural LpxC inhibitor that targets LpxC for degradation and limits lipopolysaccharide production in Enterobacteriaceae. Contrary to some studies, findings herein support that LapB remains in complex instead of dissociating from its presumed negative regulator, PbgA/YejM/LapC, under conditions where LpxC proteolysis is enhanced. Advanced comprehension of this critical protein-lipid signaling network will lead to future development and refinement of small molecules that can specifically interfere.

## INTRODUCTION

Gram-negative bacteria generate an outer membrane (OM) permeability barrier that protects against antibiotics, noxious compounds, and immune systems (1, 2). The bilayer uniquely consists of glycerophospholipids (GPL) within the inner leaflet and lipopolysaccharides (LPS) within the outer leaflet (3, 4). Bacteria must maintain their OM-lipid asymmetry and control their LPS abundance to withstand stress and repel hazards in their environment (1, 5–7). LPS biogenesis is a highly regulated, energy-dependent, and nutrient-consuming process that demands the use of shared substrates. Accordingly, bacteria evolved and adapted proteins to regulate the level and activity of key rate-limiting enzymes, which enables them to modify their envelope to fit their surroundings (8–10). These strategies prevent nutrient depletion and allow the appropriation of finite resources, which facilitates bacterial growth and division and improves bacterial endurance and survival.

LpxC is a conserved uridine diphosphate-3-*O*-acyl-N-acetylglucosamine (GlcNAc) deacetylase that catalyzes the rate-limiting step of LPS biosynthesis in many Gram-negative bacteria (11–13). LPS biosynthesis consumes R-3-hydroxy-myristate and GlcNAc, which are dually necessary for GPL and peptidoglycan (PG) biosynthesis, respectively (14). Cell envelope biogenesis demands the synchronized regulation of these pathways for bacteria to effectively produce a protective barrier. LpxC overexpression causes LPS overproduction and attenuates OM assembly, reduces barrier integrity, and restricts cellular growth (8, 15–18). Likewise, LpxC inhibition and depletion diminish bacterial LPS production, perturb OM biogenesis, increase barrier permeability, and impair bacterial growth (18, 19). Consequently, LpxC is a tightly regulated and essential enzyme for most species. Since homologs are absent from mammals, LpxC is a target for substrate-mimetic inhibitors as antibiotics (19–23).

**Fig 1:**
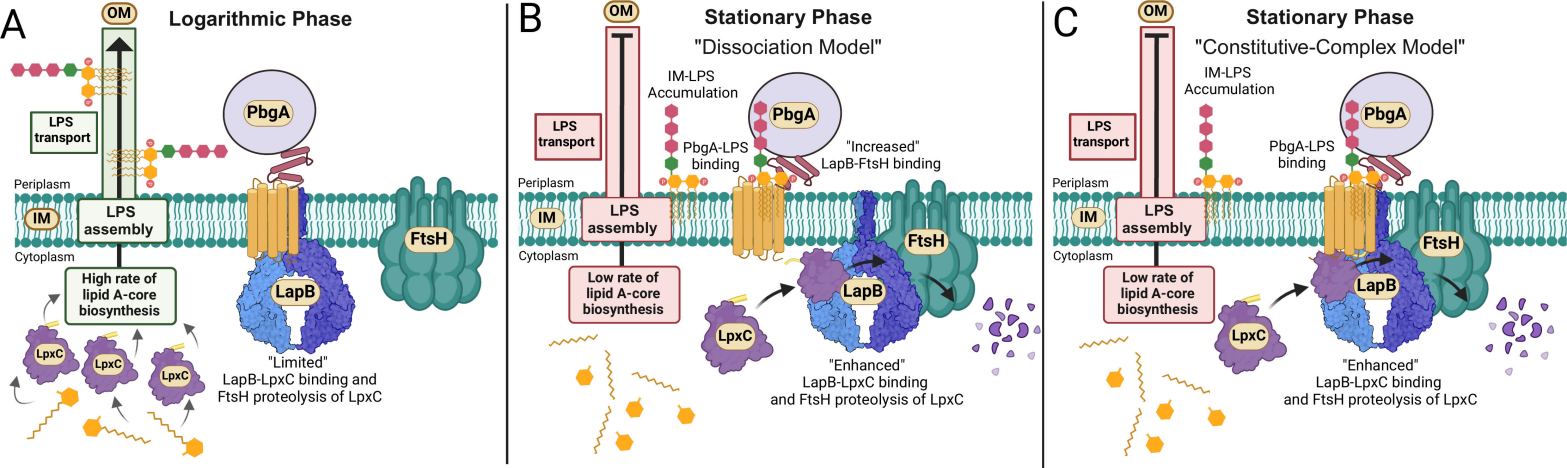
Models of enterobacterial LPS regulation involving the PbgA-LapB complex and LPS sensing at the IM. (**A**) Prevailing models suppose that when nutrients are abundant and bacteria are in logarithmic-phase growth (log phase), the coupled processes of bacterial LPS synthesis and transport proceed rapidly and PbgA is unbound to LPS molecules yet bound to LapB. When unbound to LPS, the PbgA-LapB complex is conformationally restricted for LpxC and FtsH binding, which prevents LapB from driving LpxC proteolysis. Two conflicting models exist for PbgA-LapB interplay when the bacterial environment deteriorates. (**B**) In response to limiting nutrients, such as during stationary-phase growth, PbgA signals for LPS downregulation by binding LPS molecules that accumulate on the periplasm-IM interface. In the “dissociation model,” PbgA-LPS binding releases LapB so that LapB can bind LpxC and FtsH to promote LpxC proteolysis. (**C**) We present data to contradict the “dissociation model” and instead provide evidence for what we term the “constitutive-complex model,” whereby PbgA-LapB forms a complex during both log- and stationary-phase growth that differentially binds LpxC to facilitate regulated proteolysis.

Enterobacteriaceae evolved a system of essential inner membrane (IM) proteins that interact with one another to regulate LpxC via proteolysis. This regulatory strategy allows bacteria to control OM-LPS abundance and balance rates of GPL and PG biosynthesis with LPS biosynthesis in response to fluctuations in the environment. Lps assembly protein B (LapB/YciM) is a stress-induced single-pass IM protein identified in *Escherichia coli* (*E. coli*) that binds LpxC and the protease, FtsH, and controls LpxC delivery to FtsH to enhance LpxC proteolysis (18, 24–30). PhoPQ-barrier gene A (PbgA/LapC/YejM), named for its functional involvement during the activation of the PhoPQ two-component virulence regulators in *Salmonella enterica* serovar Typhimurium (*S*. Typhimurium), is a multipass IM protein that binds LapB’s transmembrane (TM) (31). PbgA carriers a large periplasmic domain (PD) and interfacial region that lies between the PD and transmembrane region, which binds LPS molecules and controls whether LapB’s cytosolic domain is able to enhance LpxC proteolysis (Fig. 1). Several studies support disparate models of how PbgA and LapB operate within this regulatory pathway (26, 32–37).

When nutrients are abundant, bacterial LPS synthesis proceeds rapidly and PbgA is unbound to LPS molecules and in complex with LapB (Fig. 1A). Under these conditions, LpxC is stable and PbgA-LapB is conformationally restricted from binding to LpxC and FtsH, and this prevents LapB from promoting LpxC proteolysis. When nutrients are limited, growth restriction stalls LPS assembly at the OM, which causes LPS molecules to accumulate at the periplasm-IM interface (Fig. 1B). In the “dissociation model,” PbgA binds these LPS molecules causing LapB to dissociate from PbgA, which frees LapB to bind LpxC and FtsH and enhance LpxC proteolysis (26–28, 34, 36). By contrast, the “constitutive-complex model” predicts that the PbgA-LapB complex does not dissociate when nutrients become limiting. Instead, growth restriction results in PbgA-LPS interactions at the periplasm-IM interface that promote an unrestricted conformation of PbgA-LapB that permits LapB binding to LpxC and FtsH in order to drive LpxC proteolysis (Fig. 1C) (33, 35). Conflicting data exist to support these models, and the mechanisms are not fully resolved.

Here, we sought to determine how the facultative intracellular pathogen, *S*. Typhimurium, uses PbgA-LapB to govern LpxC proteolysis and control LPS biosynthesis at the transition from log to stationary phase. The goal was to define the biological and biochemical function of LapB toward binding LpxC and regulating LpxC levels in these bacteria. Our findings support that PbgA and LapB interact in both growth phases and that PbgA’s interaction with LapB and LapB’s interaction with LpxC are each enriched in stationary-phase salmonellae. Likewise, as shown recently for *E. coli*, PbgA and LpxC interact in *S*. Typhimurium (28). In summary, PbgA-LapB is a keystone regulator of enterobacterial cell envelope biogenesis that impinges on LpxC in a manner that could inform new and existing antimicrobials that target LPS biosynthesis enzymes.

## RESULTS

### *S*. Typhimurium relies on *lapB* to limit LpxC and lipid A-core abundance, which promotes OM barrier integrity and supports cellular growth

To determine whether LapB regulates LpxC proteolysis and LPS biosynthesis, we generated a *lapB* deletion-insertion mutant in our *S*. Typhimurium 14028s genotype encoding the *wza-lacZ* transcriptional reporter of OM integrity (31, 32, 37, 38). The *wza* promoter is induced by the enterobacterial Iga-Rcs signaling cascade, which controls colanic-acid capsule biosynthesis (39–41). The OM sensor lipoprotein, RcsF, detects perturbations in OM integrity, relocates to the IM, and alleviates Iga-mediated repression of the RcsABCD sensor-histidine kinase/response-regulator system, which activates the *wza* operon (39–43). RcsF-dependent *wza-lacZ* activation can be visualized and quantified to assess variations in OM barrier integrity (31, 32, 37, 38). Consistent with *lapB* promoting bacterial growth and OM integrity, pinpoint-sized blue colonies of *lapB* mutant (Δ*lapB*) *S*. Typhimurium were isolated at 30°C on nutrient-rich indicator agar (Fig. S1A and B).

**Fig 2:**
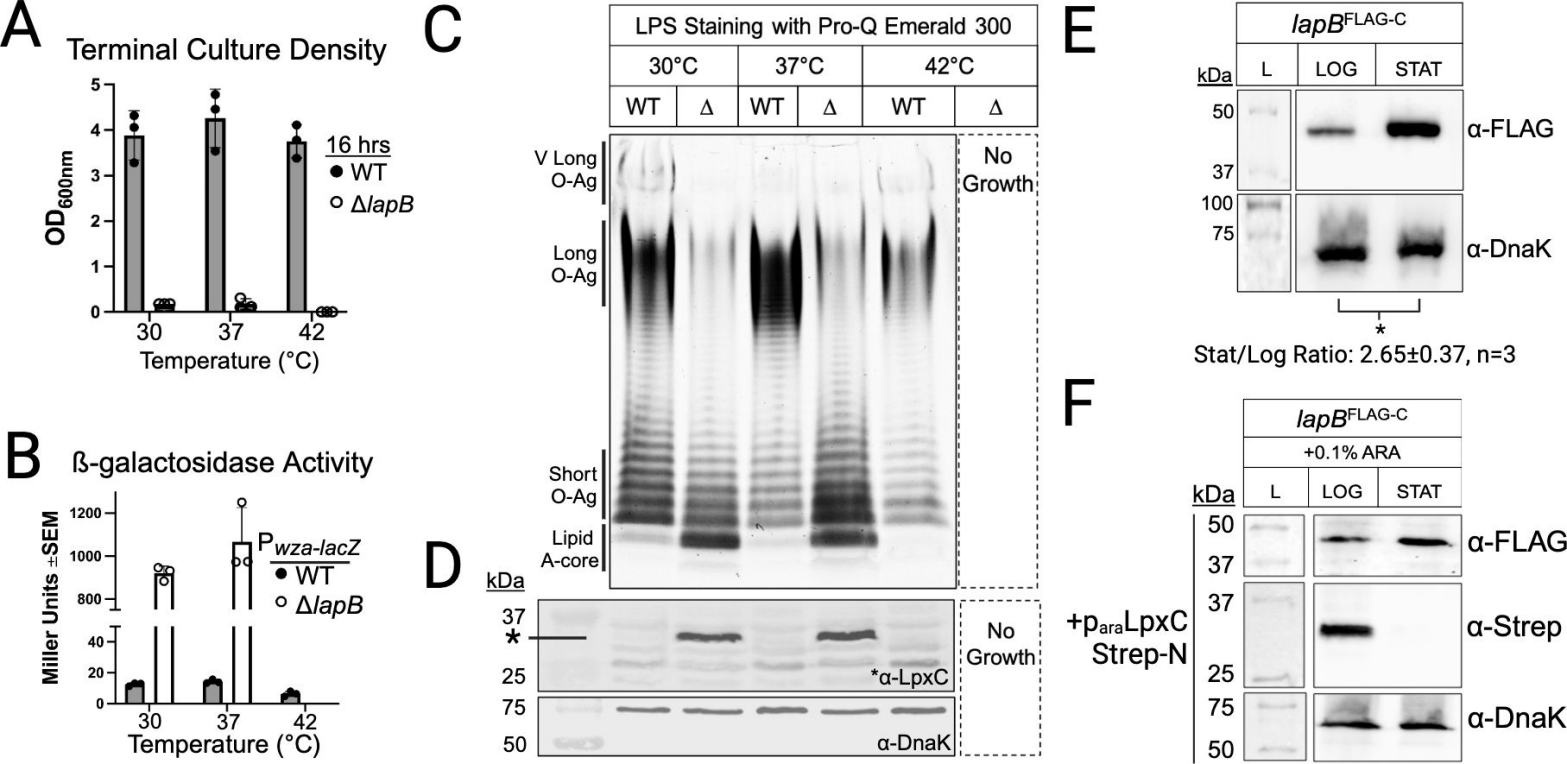
LapB levels increase in the stationary phase while LpxC levels decrease, and *lapB* is necessary for *S*. Typhimurium growth, OM integrity, and LPS composition. (**A**) *S*. Typhimurium *lapB* is required for growth and thermo-tolerance in nutrient-rich broth media. (**B**) Δ*lapB* activates the *wza-lacZ* gene reporter of OM integrity as measured by β-galactosidase activity and quantified as Miller units. (**C**) *lapB* mutants accumulate lipid A-core molecules and are depleted for LPS molecules with **L** and VL O-antigens relative to the wild type when cultured to the stationary phase. LPS samples were extracted from normalized cell culture densities [optical density (OD_600_)]. (**D**) Immunoblot depicting the requirement of *lapB* to limit 34 kDa LpxC levels (α-LpxC, upper panel, demarcated by asterisk) in comparison to a loading control (α-DnaK, lower panel). (**E**) LapB^Flag-C^ levels were monitored in the log and stationary growth phases and semi-quantified using an α-FLAG antibody (top panel) in comparison to a loading control (α-DnaK, bottom panel). The increase in LapB^Flag-C^ levels in stationary-phase bacteria versus log-phase bacteria was statistically significant (^*^P = 0.037) by paired Student’s *t*-test. (**F**) LpxC expression decreases as LapB expression increases during the log-to-stationary phase growth transition. Immunoblots of *lapB^FLAG-C^* from whole-cell lysates of bacteria induced (+0.1% ARA) to express a plasmid-borne copy of an amino-terminal streptavidin tagged LpxC (LpxC^Strep-N^). OD_600_ readings were used to define the log phase as OD_600_ of 0.6–0.8 and the stationary phase as the culture density obtained after incubation at 37°C for 16 h. Data are reported as an average of three biological replicates and represent the standard error of the mean (±SEM).

Growth and thermotolerance were evaluated by culturing Δ*lapB* in a nutrient-rich broth medium at 30°C, 37°C, and 42°C alongside the wild type. Comparatively, Δ*lapB* grew to very low densities at 30°C and 37°C and did not grow at 42°C (Fig. 2A). *wza-lacZ* levels were significantly greater for the mutant than for the wild type, indicating a defect in OM integrity (Fig. 2B). Isolation and visualization of LPS molecules revealed several changes in the mutant cell envelope (Fig. 2C). For instance*,* Δ*lapB* produced qualitatively greater amounts of lipid A-core molecules, which are LPS precursors devoid of O-polysaccharides that are also referred to as O-antigens. Qualitatively, Δ*lapB* routinely produced lesser amounts of LPS molecules with long (L, 16–35 repeating units) and very long (VL, >100) O-antigens (Fig. 2C). The LPS changes were accompanied by a heightened level of LpxC in Δ*lapB* relative to the wild type (Fig. 2C and D). The data support existing *E. coli* studies, whereby LapB is a negative regulator of the cellular abundance of LpxC and lipid A-core, which is essential for *S*. Typhimurium LPS biogenesis, OM integrity, and growth.

### *S*. Typhimurium concomitantly increases the level of LapB and decreases the level of LpxC during the log-to-stationary phase transition

*S*. Typhimurium relies on *pbgA* and *lapB* to regulate LPS biosynthesis at the log-to-stationary phase transition in nutrient-rich broth media (32, 37). We suspected that salmonellae increase LapB levels in the stationary phase to enhance LpxC degradation. To measure LapB, we fused an epitope tag to the native allele on the chromosome, *lapB^Flag-C^* (44). Indeed, *S*. Typhimurium produced significantly greater levels of LapB in the stationary phase than in the log phase (2.65 ± 0.37 fold change, *P* = 0.037) (Fig. 2E; Tables S1 and S2). Attempts to tag the C-terminus of LpxC on the chromosome were not successful. Likewise, although we could consistently show an accumulation of LpxC in the Δ*lapB* mutant versus the wild type, severe cross-reactivity and inconsistency with commercial LpxC antibodies and a generally low abundance of native LpxC in wild-type *S*. Typhimurium limited our ability to monitor variations between growth phases (Fig. 2D)(37). Alternatively, we used a plasmid and fused a streptavidin tag to the N-terminus of LpxC and cloned the allele behind an arabinose-inducible promoter (45). LpxC expression was induced in early-log-phase cells, and the level was assessed for log-phase (OD_600_ 0.6–0.8) and stationary-phase (OD_600_ 3.5–3.8) bacteria. Log-phase bacteria produced a detectable amount of LpxC while stationary-phase bacteria consistently produced a severely diminished and barely detectable amount of the enzyme (Fig. 2F). The data support the hypothesis that in the stationary phase *S*. Typhimurium increases the level of LapB to decrease the level of LpxC.

### Similar to *E. coli*, *lapB*’s essential function in *S*. Typhimurium is to limit the rate of lipid A-core biosynthesis

Extended culturing of Δ*lapB* led to suppressor isolates with larger mucoid light blue and non-mucoid white colony morphotypes among the pinpoint blue-colony parental morphotype. Genome sequencing identified non-synonymous polymorphisms in *lpxA* and *lpxC,* which encode the first two enzymes for lipid A-core biosynthesis (Table S3) (46). Phenotypic measurements indicated that the LpxA and LpxC substitutions restored growth and Rcs signaling defects but did not restore the aberrant increase in LpxC levels observed for the Δ*lapB* parental genotype (Fig. S2A through C). However, the lipid A-core, L-LPS, and VL-LPS levels were largely repaired in the suppressor isolates compared to Δ*lapB* (Fig. S2C). Hence, the LpxA and LpxC mutations restore LPS biogenesis but do not alter the level of LpxC (Fig. S2A through C). The data are consistent with *S*. Typhimurium requiring *lapB* to limit lipid A-core biosynthesis to grow and divide in laboratory media.

### *S*. Typhimurium LapB promotes cellular growth, LpxC and LPS regulation, and virulence

**Fig 3:**
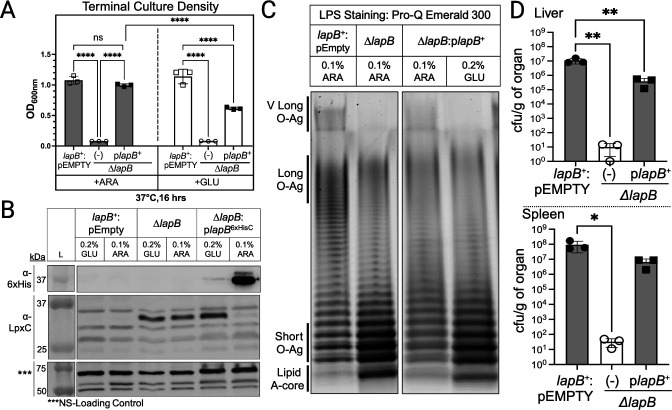
Transcomplementation of the *lapB* deletion mutant with LapB restores *S*. Typhimurium growth, LpxC levels, and LPS composition. (**A**) Arabinose induction of LapB *in trans* is sufficient to restore the *lapB* mutant growth defect, while glucose repression results in an intermediate growth phenotype. Bacterial growth was assessed by measuring the terminal OD_600_ of stationary-phase bacteria. Statistical significance was calculated using one-way ANOVA followed by Tukey’s multiple comparisons test comparing the mean of each column with the mean of every column (^*^P < 0.0332, ^**^P < 0.0021, ^***^P < 0.0002, and ^****^P < 0.0001) (*n* = 3, ±SEM). (**B**) LapB induction (0.1% ARA) increases LapB expression levels and decreases LpxC levels while repressing LapB levels (0.2% GLU) increases LpxC levels. For reference, the estimated size of LapB is 44 kDa, LpxC is 34 kDa, and the cross-reactive non-specific protein band routinely detected by the polyclonal LpxC antisera is 70 kDa (denoted by ***, served as loading control). (**C**) LapB induction (0.1% ARA) restores the levels of lipid A-core and L- and VL-LPS molecules to *lapB* mutant *S*. Typhimurium, while glucose repression (0.2% GLU) resembles the LPS profile of the *lapB* mutant without the vector. (**D**) Female and male C57BL/6J mice were intraperitoneally injected with ~5 × 10^5^ cfu of the wild type, the *lapB* mutant, and the transcomplemented *lapB* mutant post-culturing for 16 h at 37°C in Luria-Bertani/lysogeny broth (LB). After 2 days post-infection, the mice were euthanized, and colony counts were enumerated from liver and spleen homogenates. Data are shown as the mean number of cfu per gram of liver ±SEM and spleen ±SEM. Each genotype was assessed in at least three mice (*n* = 3). Statistical significance was calculated using one-way ANOVA followed by Dunnett’s multiple comparisons test using *lapB*^+^:pEMPTY as a control group (^*^P < 0.0332, ^**^P < 0.0021).

To study LapB and assess structure-function relationships, we developed a conditional *lapB* mutant. *lapB* induction in Δ*lapB* increased the level of LapB, reduced the level of LpxC, returned LPS composition to that of wild type, and increased bacterial growth (Fig. 3A through C). By contrast, *lapB* repression reduced the level of LapB, increased the level of LpxC, increased the level of lipid-A core, and caused a modest yet significant growth defect (Fig. 3A through C). Therefore, limiting the level of LapB by repressing the plasmid-borne copy in the Δ*lapB* mutant partially recapitulates the phenotypes of the deletion mutant but does not recapture the severe growth defect. Bacterial growth is, therefore, sustained by a low level of LapB, while stress-related activities demand a higher level.

Although *S*. Typhimurium Δ*lapB* mutants are severely defective for growth *in vitro*, we inquired whether our transcomplementation approach was sufficient to restore Δ*lapB* mutant virulence defects. Mice were inoculated with equivalent (~2.5 × 10^5^ cfu) amounts of wild type, Δ*lapB* mutant, and Δ*lapB* mutant carrying LapB *in trans*. The presence of LapB on the plasmid restored the severe growth and survival defect of the Δ*lapB* mutant during bacteremia and significantly increased the bacterial burden in the mouse livers and spleens albeit not statistically to the level of wild type (Fig. 3D). Therefore, *S*. Typhimurium requires LapB for growth and survival while causing bacteremia in mice, and the complementation approach can be used to test LapB residue involvement in LapB mechanisms that contribute to bacterial pathogenesis.

### Consistent with previous work, LapB’s TM is necessary for LapB’s function toward regulating LpxC and promoting bacterial growth

**Fig 4:**
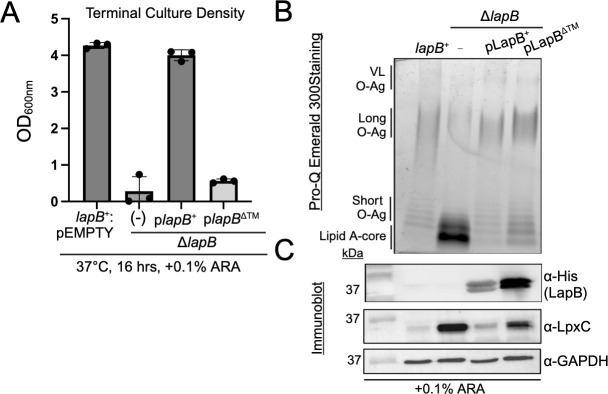
*S*. Typhimurium requires LapB’s transmembrane to limit LpxC expression, but overexpression of the cytosolic domain is sufficient to limit lipid A-core biosynthesis and alter LPS production. (**A**) Terminal liquid culture density (16 h, 37°C, LB, +0.1% ARA) of *lapB^+^*:pEMPTY, ∆*lapB* mutant, a *lapB* mutant transcomplemented with full-length LapB (*lapB^+^,* FL), or transmembrane-deficient LapB (*lapB*^∆TM^, ∆TM) was recorded and analyzed (*n* = 3, ±SEM). (**B**) LPS analysis comparing wild-type, ∆*lapB*, ∆*lapB*: p*lapB^+^*, ∆*lapB*: p*lapB*^∆TM^. LapB and LpxC immunoblot derived from whole-cell lysates. α-His antibody was used to verify LapB presence alongside polyclonal α-LpxC in comparison to α-GAPDH as a loading control.

LapB contains an N-terminal signal sequence that comprises a TM segment. The TM orients a short helical extension into the cytosol followed by nine tetratricopeptide repeats (TPRs) and a C-terminal rubredoxin (Rdx) domain (Fig. S3A) (24, 47). We then asked whether *S*. Typhimurium requires LapB’s TM to function by removing it from the plasmid-encoded allele. Inducing LapB^∆TM^ in the Δ*lapB* mutant led to an increase in the level of LapB but did not lead to a reduction in the level of LpxC nor did it restore the growth defect of the parental genotype relative to inducing wild-type LapB^+^ (Fig. 4A through C; Tables S1 and S2). Inducing LapB^+^ and LapB^∆TM^ resulted in qualitatively similar decreases in the level of lipid A-core and increases in the level of L- and VL-LPS molecules compared to the deletion mutant without the plasmids (Fig. 4B). We surmise that *S*. Typhimurium requires LapB’s TM to limit LpxC abundance, and LapB^∆TM^ overproduction is partly sufficient to restore lipid A-core biosynthesis and LPS biogenesis of the deletion mutant under these conditions.

### Overexpression of LapB’s cytosolic domain increases the level of LpxC, amount of Rcs signaling activity, and antimicrobial sensitivity of salmonellae

**Fig 5:**
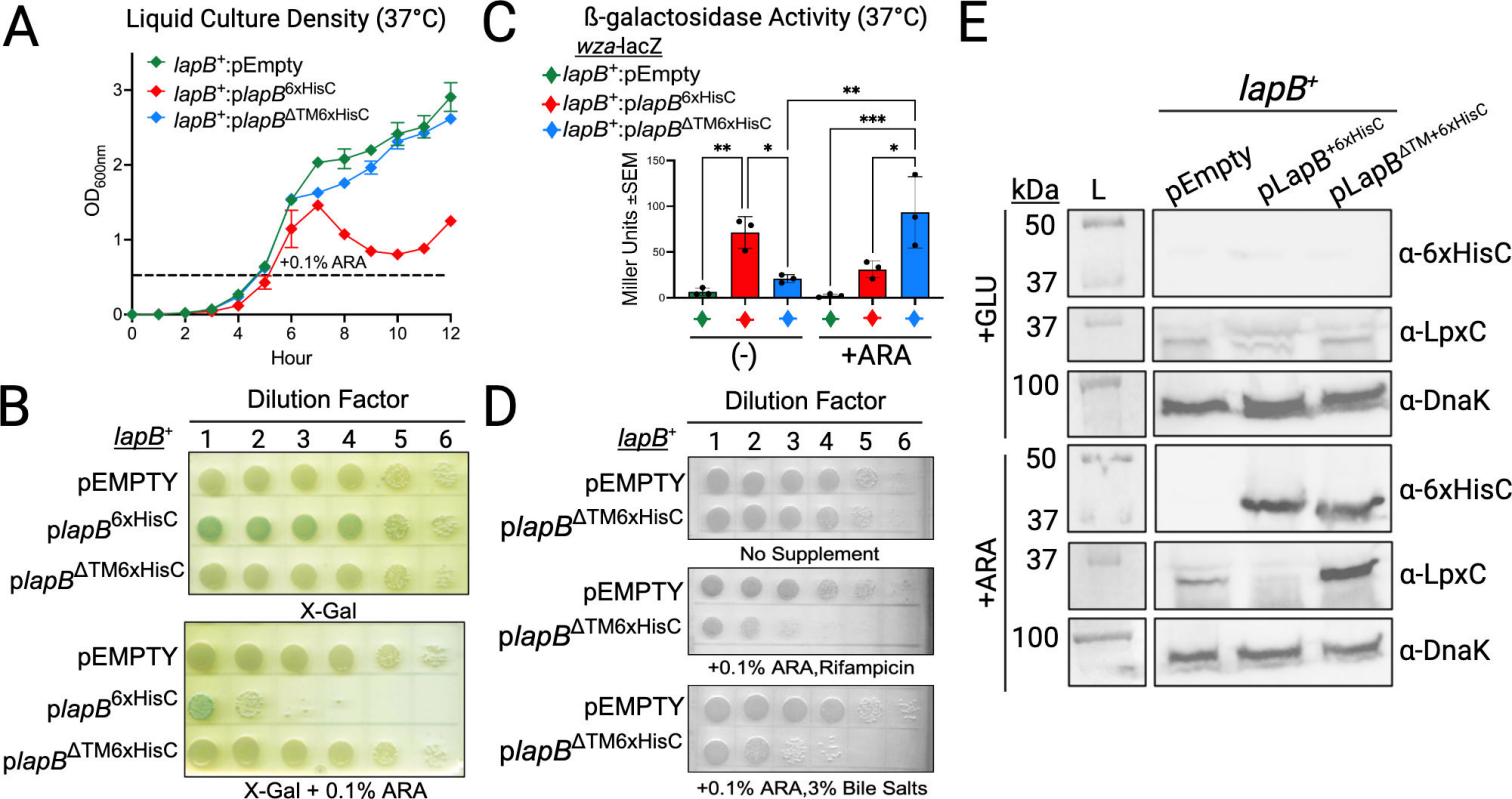
LapB overexpression diminishes LpxC levels and perturbs *S*. Typhimurium growth, while overexpressing LapB without the TM segment (LapB^ΔTM^) causes antimicrobial resistance to decrease and LpxC to accumulate. (**A**) LapB induction in wild-type *S*. Typhimurium (*lapB*^+^) limits bacterial growth in a manner that requires the TM segment. The *lapB*^+^:pEMPTY and *lapB*^+^ bacteria carrying a plasmid-borne copy of wild type (WT) (pLapB^6xHisC^) or mutant (pLapB^∆TM6xHisC^) were cultured to the log phase, 0.1% arabinose was added to the media, and bacteria growth was monitored as a function of time. (**B**) Arabinose induction of *lapB*^+^:pLapB^6xHisC^ limits bacterial growth on solidified LB agar while *lapB*^+^:pLapB^ΔTM6xHisC^ does not. (**C**) Overexpressing LapB^6xHisC^ or LapB^ΔTM6xHisC^ activates the *wza-lacZ* reporter of OM integrity damage as measured by β-galactosidase activity and quantified as Miller units. Statistical significance was calculated via ordinary one-way ANOVA followed by Tukey’s multiple comparisons test (^*^P < 0.0332, ^**^P < 0.0021, ^***^P < 0.002, and ^****^P < 0.0001) (*n* = 3, ±SEM). (**D**) Induction of pLapB^∆TM6xHisC^ increases *S*. Typhimurium sensitivity to rifampin and bile salts. (**E**) Induction of pLapB^6xHisC^ elevates LapB levels, but no detectable LpxC was present, while induction of pLapB^∆TM6xHisC^ results in elevated levels of LapB and LpxC (*n* = 3).

The overproduction of LapB retards bacterial growth and limits the level of LpxC (18, 24, 25). Therefore, instead of using the complementation plasmids to rescue the *lapB* mutant, we used them to test whether increasing the level of LapB^+^ or LapB^∆TM^ would alter the level of LpxC, impair growth, or damage the barrier of wild-type *S*. Typhimurium. For this approach, we measured bacterial sensitivity to membrane-permeating antimicrobial agents including rifampin and bile salts. Indeed, increasing the level of LapB diminished the level of LpxC, increased the amount of Rcs signaling activity, and attenuated bacterial growth (Fig. 5A through E). By contrast, increasing the level of LapB^ΔTM^ increased the level of LpxC, increased the amount of Rcs signaling activity, and decreased *S*. Typhimurium resistance to rifampin and bile salts but did not impair bacterial growth (Fig. 5A through E). The results are consistent with a high level of LapB^∆TM^ impacting native LapB’s ability to function toward enhancing LpxC proteolysis (26). The prevailing evidence supported that LapB binds LpxC to limit LpxC abundance, so we began to test this prediction.

### LapB^ΔTM^ forms a homoligomeric complex that binds LpxC^Δ293-305^

**Fig 6:**
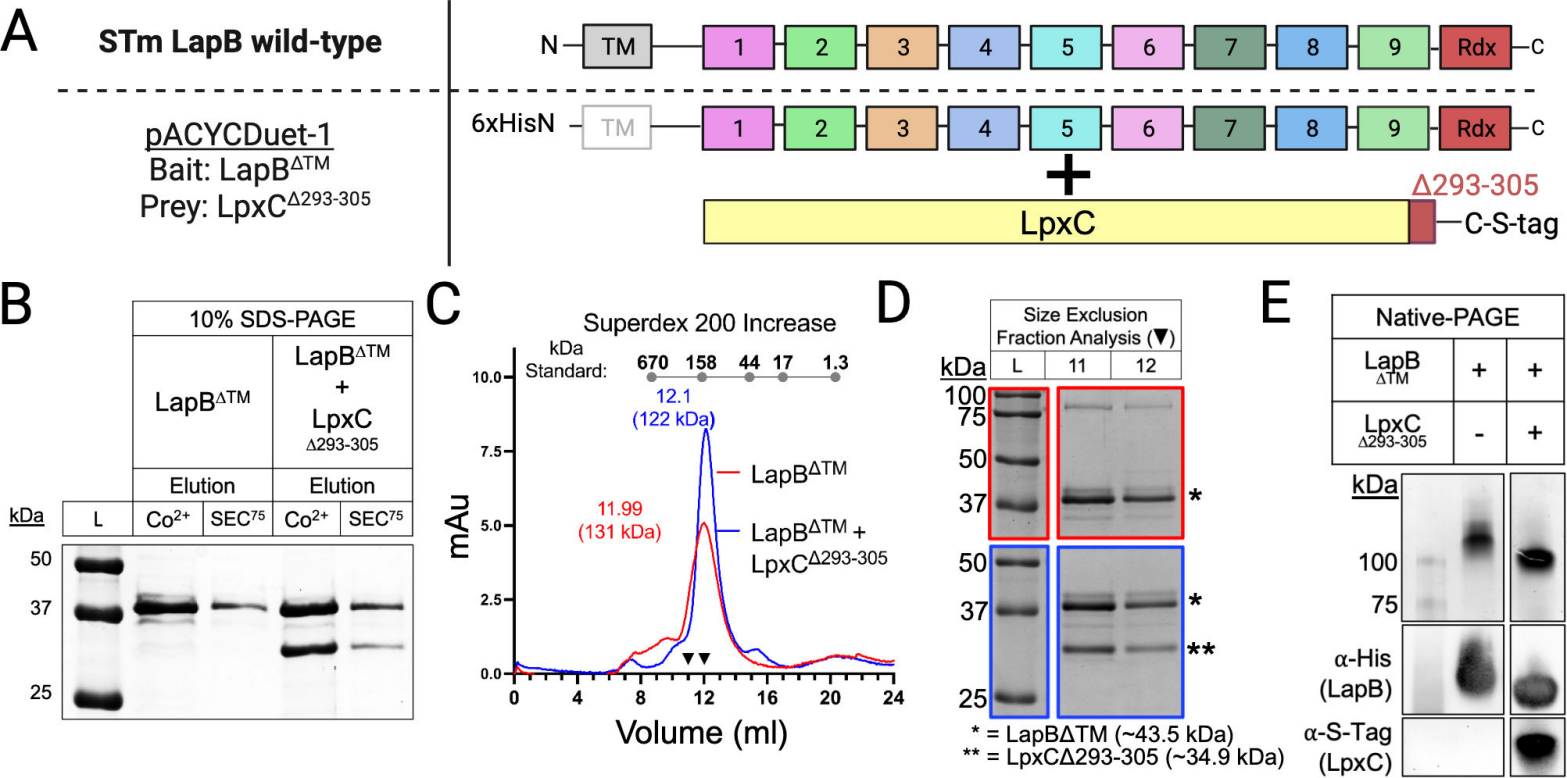
LapB alone forms a homotrimeric complex, and LapB-LpxC forms a heterotrimeric complex of one LpxC molecule and two LapB molecules. (**A**) Linear schematic of the engineered proteins encoded onto pACYCDuet-1, co-overexpressed, and purified. The LapB TM was removed to avoid detergent use and replaced with an N-terminal polyhistidine tag (^6xHisN^LapB^∆TM^). The LpxC C-terminal tail was removed to increase protein stability and replaced with an S-Tag (LpxC^∆293-305-S-Tag^). (**B**) LapB and LpxC co-overexpression followed by affinity purification and gel filtration results in co-elution of LapB and LpxC. SDS-PAGE analysis of the elution fractions from a two-step affinity purification using cobalt resin (Co^2+^) and gel filtration analysis with a Superdex 75 column (SEC^75^). (**C**) LapB forms a homotrimeric complex while LapB-LpxC forms a heterotrimeric complex of two LapB molecules and one LpxC molecule. Calibrated gel filtration chromatograms of the purified LapB or the LapB-LpxC complexes followed by (**D**) SDS-PAGE analysis of the peak fractions corresponding to the 11–12-mL elution volumes. A protein standard mix consisting of bovine thyroglobulin (670 kDa), bovine gamma-globulin (158 kDa), ovalbumin (44 kDa), myoglobulin (17 kDa), and vitamin B12 (1.35 kDa) was used to generate a standard curve and estimate molecular weight (gray dotted line above the peaks in the representative chromatogram). (**E**) The LapB complex is larger than the LapB-LpxC complex. Native-PAGE analysis of 5 µg of purified LapB or co-purified LapB-LpxC. Protein presence was verified by Coomassie staining (upper images) or Western blotting (lower images) with antibodies against the affinity tags, α-His and α-S-tag.

Structural studies suggest that LapB^+^ and LapB^∆TM^ form homodimers (Fig. S3B) (26, 27, 47). Although the LapB monomers intersect near TPRs1-2, the dimer interface is within C-terminal TPRs proximal to the Rdx (27, 47). To analyze LapB-LpxC complex formation, we developed a co-overexpression and purification system. The LapB TM was replaced by a polyhistidine tag, and the C-terminal tail of LpxC was substituted for an S-tag to facilitate the detection of a stable complex (Fig. 6A) (16, 48). Co-overexpression and purification of LapB^ΔTM^ (43.5 kDa) and LpxC^Δ293-305^ (35.9 kDa) resulted in co-elution of proteins with molecular weights of roughly 44 and 36 kDa, while overexpression and purification of LapB^ΔTM^ resulted in the elution of a single protein of 44 kDa (Fig. 6B). Calibrated gel filtration showed that LapB^ΔTM^ and LapB^ΔTM^-LpxC^Δ293-305^ elute as complexes greater than 100 kDa (Fig. 6C). Specifically, LapB^ΔTM^-LpxC^Δ293-305^ eluted at 12.10 mL or 122 kDa, and LapB^ΔTM^ eluted at 11.99 mL or 131 kDa (Fig. 6D). Native-PAGE revealed that LapB^ΔTM^-LapB^ΔTM^ migrates at a slightly slower rate than LapB^ΔTM^-LpxC^Δ293-305^, yet both complexes migrate slower than the 100-kDa protein marker (Fig. 6E). Therefore, LapB oligomerizes and accommodates LpxC binding irrespective of the TM segment. Measurements here suggest that LapB-LpxC exists as a dimeric oligomer of LapB (~87 kDa) in complex with a LpxC monomer (~36 kDa) under these conditions (27, 47). Unlike the dimeric oligomerization sized previously, LapB^ΔTM^ forms trimeric oligomers (~132 kDa) in our study (Fig. 6C through E) (27, 47). Additional investigation is needed to determine the physiological significance and functional involvement of LapB’s oligomeric state, as well as the oligomeric state of the LapB-LpxC complex, in regulating in bacterial LpxC.

### LapB^ΔTM^ binds LpxC^Δ293-305^ using TPRs 2-4 near the cytosol-IM interface

**Fig 7:**
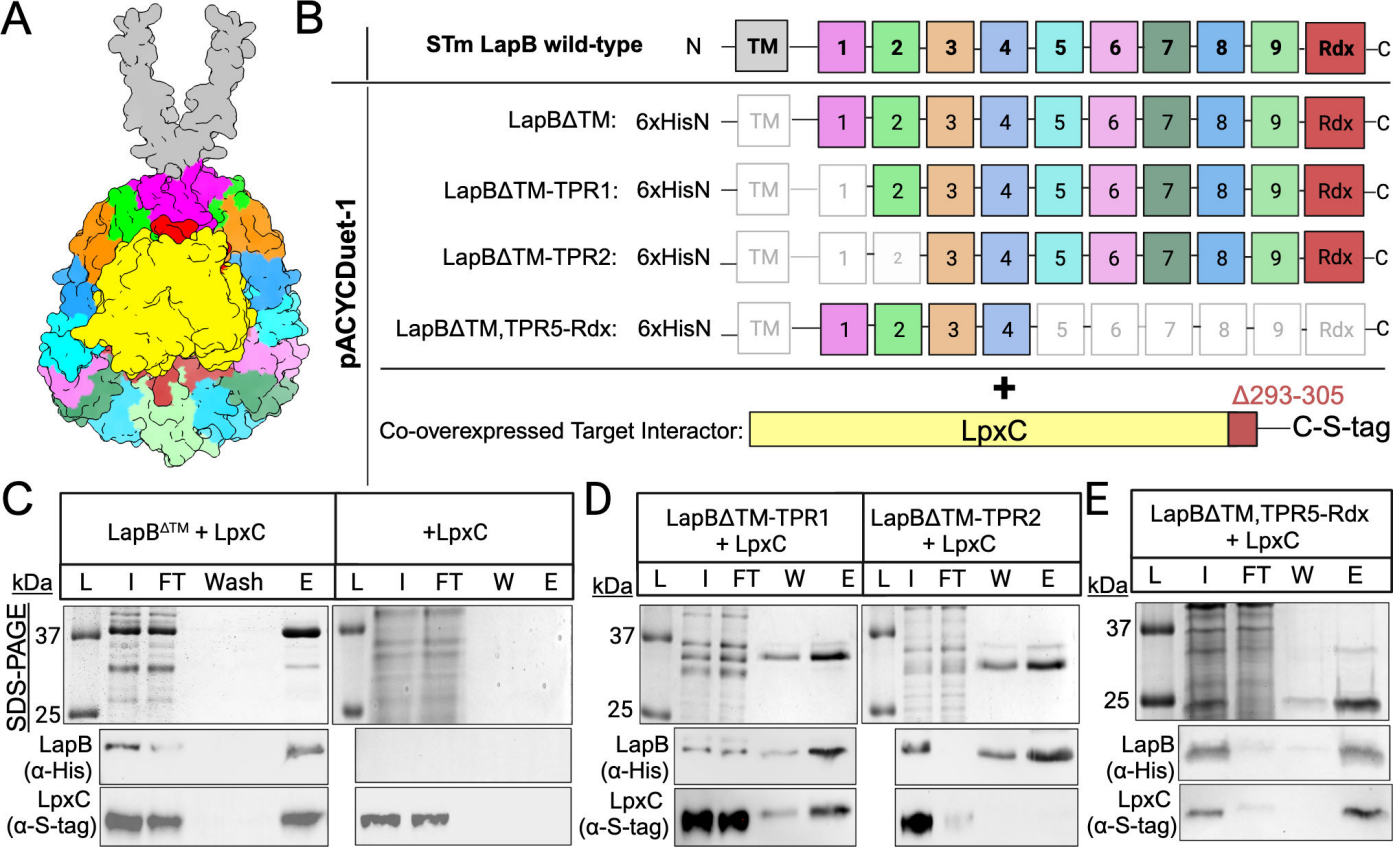
LapB binds LpxC using TPRs 2–4 proximal to the amino-terminal TM. (**A**) Surface representation of AlphaFold2 multimer-predicted LapB-LpxC complex. (**B**) Linear schematic depicting sequential amino-terminal LapB TPR deletion and carboxyl-terminal TPR-Rdx deletions used to identify the LapB-LpxC interaction interface. (**C**) LapB and LpxC co-elute following microscale affinity purification. SDS-PAGE analysis of fractions collected from the purification of ^6xHisN^LapB^∆TM^ and LpxC^Δ293-385-S-Tag^ (left panel) or LpxC^Δ293-385-S-Tag^ alone (right panel) using cobalt-coated microbeads. Sample fractions were normalized to 2.5 µg of protein and equally loaded and resolved on a 10% SDS-PAGE gel and immunoblotted using affinity tag-specific antibodies: α-His for LapB and α-S-tag for LpxC. [L] refers to ladder, [I] refers to input, [FT] refers to flow-through, [W] refers to wash, and [E] refers to elution. Single expression and purification of LpxC^Δ293-385-S-Tag^ were used as a control to exclude non-specific binding to the magnetic beads (right panel). (**D**) LapB TPR1 is not required for the interaction with LpxC, while TPR2 is necessary. Representative co-purification of LapB^∆TM-TPR1^ (left panel) and LapB^∆TM-TPR2^ (right panel). (**E**) LapB TPRs 1–4 are sufficient for LapB to interact with LpxC while TPRs 5–9 and the Rdx are dispensable. Each purification is representative of one of three biological replicates.

*E. coli* LapB interacts with other critical enzymes for LPS and GPL biosynthesis (25, 28). However, the LapB regions and residues that mediate these interactions are not known. Interestingly, multiple AlphaFold2 LapB-LpxC prediction models orient LpxC toward the amino-terminal concave face near TPRs 2–4 (Fig. 7A; Table S4) (49). Numerous polymorphisms have been identified in TPRs 2–4 that restore LpxC levels and LPS biosynthesis defects for various *E. coli*, *S*. Typhimurium, and *Klebsiella pneumoniae* genotypes (Fig. S4A) (25, 32, 34, 35, 50, 51). However, the mechanism and role of these residues and TPRs have not been elucidated. We hypothesized that LapB binds LpxC through TPRs 2–4 (Fig. 7A; Fig. S4B).

Amino-terminal TPRs were sequentially deleted from LapB^ΔTM^ and tested for LpxC^Δ293-305^ co-purification in the dual expression system (Fig. 7B; Tables S1 and S2). Co-overexpression of LapB and LpxC and purification and elution of LapB resulted in LpxC co-elution, supporting an interaction. Overexpression of LpxC alone did not result in LpxC co-elution when affinity-purified according to the preparation of LapB, which indicated a lack of non-specific LpxC retention (Fig. 7C). Removing LapB TPR1 did not reduce LpxC co-elution, while removing LapB TPRs 1–2 diminished co-elution (Fig. 7D). Therefore, TPR2 facilitates LpxC binding. Next, TPRs 5–9 and the Rdx were removed to ask whether TPRs 1–4 were sufficient to co-elute LpxC. Remarkably, LapB TPRs 1–4 retained the ability to co-purify with LpxC (Fig. 7E). Hence, LapB binds LpxC in part using residues within TPR2. Residues within TPRs 3 and 4 are likely involved (Fig. S4). Our data suggests that the TM, TPR1, TPRs 5–9, and the Rdx are not required for LapB-LpxC complex formation under these conditions. However, our data do not exclude that LpxC interacts with these other features of LapB in the bacterium.

### The PbgA-LapB interaction is detectable in log phase and enriched in stationary-phase bacteria

**Fig 8:**
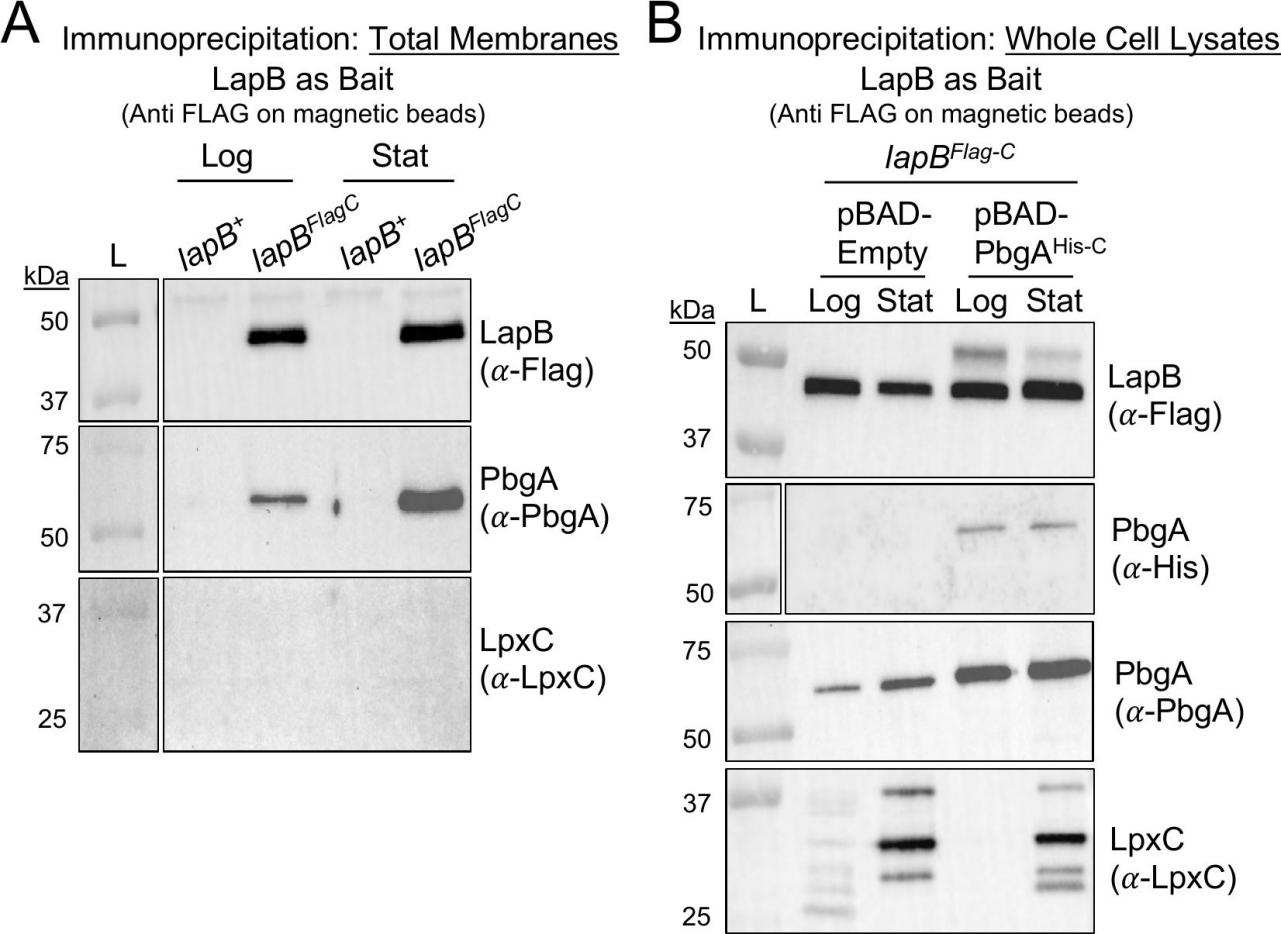
LapB interacts with PbgA in log- and stationary-phase bacteria and PbgA-LapB as well as LapB-LpxC interactions are enriched in stationary-phase salmonellae. (**A**) Immunoprecipitation of LapB from the membranes of log- and stationary-phase *S*. Typhimurium results in the co-elution of PbgA, not LpxC. Representative immunoprecipitation assay with anti-Flag loaded magnetic beads. Equal amounts of isolated membranes from *S*. Typhimurium *lapB^+^* or *lapB^Flag-C^* cultured to either the log or stationary phase were incubated with the beads, washed, and eluted. Fifteen microliters of the elution fraction was analyzed by SDS-PAGE and immunoblotting. The following proteins were examined: LapB^FLAG-C^ (47 kDa) using anti-Flag monoclonal antibody, LpxC (34 kDa) using anti-LpxC polyclonal antibody, and PbgA (67 kDa) using anti-PbgA polyclonal antibody. (**B**) Immunoprecipitation of LapB from *S*. Typhimurium whole-cell lysates collected from log- and stationary-phase bacteria results in the co-elution of PbgA in both growth phases, while both PbgA and LpxC are enriched in the elution fractions of stationary-phase bacteria. A representative immunoprecipitation assay with anti-Flag loaded magnetic beads from whole-cell lysates was prepared from 0.1% arabinose-supplemented cultures of *lapB^Flag-C^* harboring a plasmid-borne copy of PbgA^6xHis-C^ or an empty vector, which had been harvested in the log or stationary growth phase. Equal amounts of bacterial lysates were incubated with the magnetic beads, washed, and eluted. Fifteen microliters of the elution fraction was analyzed by SDS-PAGE and Western blotting. The same proteins were assessed as in panel (**A**) apart from PbgA^6xHis-C^ (68 kDa), which was visualized using an anti-His monoclonal antibody. The lines tooled around the marker lanes are drawn to distinguish between two different image files of the same membrane. The left most image file of the ladder is the colorimetric image. In some instances, the secondary antibody interacts with the protein markers in the ladder; therefore, the chemiluminescent image alone cannot always be used.

LapB interacts with LpxC through specific TPRs and with PbgA and FtsH through the TMs (Fig. 6 and 7) (26, 28, 33, 34). PbgA does not directly bind to FtsH (28, 33, 34). To determine whether LapB binds PbgA and LpxC as part of regulating LpxC proteolysis, we immunoprecipitated LapB^Flag-C^ from the isolated membranes of log- and stationary-phase bacteria. Membranes of bacteria encoding untagged LapB^+^ or tagged LapB^Flag-C^ were incubated with beads pre-loaded with anti-Flag antibodies. Immunoblotting the elutions revealed the ~47 kDa LapB^Flag-C^ protein from the *lapB^Flag-^*^C^ but not the *lapB^+^ S*. Typhimurium, which established antibody specificity (Fig. 8A). Consistent with LapB forming a constitutive complex with PbgA, antisera detected the native 67 kDa protein in the LapB^Flag-C^ elution fractions from both the log- and stationary-phase bacteria (Fig. 1A and 8A). Furthermore, the abundance of PbgA that co-eluted with LapB^Flag-C^ was consistently greater in the stationary-phase bacterial extracts than in the log-phase bacterial extracts. Importantly, the level of LapB^Flag-C^ that eluted did not vary between the growth phases (Fig. 8A). Unlike PbgA, LpxC was not detected in the LapB^Flag-C^ elution fractions in either growth phase (Fig. 8A). Therefore, PbgA and LapB interact in log- and stationary-phase *S*. Typhimurium; however, the LapB-LpxC could not be captured using this approach.

### The LapB-LpxC interaction is enriched in stationary-phase bacteria

Mounting data supported that LapB binds LpxC. As an alternative, we harvested whole-cell lysates instead of membranes and asked whether increasing the level of PbgA influenced LapB’s ability to precipitate PbgA or LpxC. Based upon the accepted model, PbgA overexpression in log-phase bacteria should diminish LapB’s ability to bind LpxC (Fig. 1A). Therefore, *lapB^Flag-C^* was transformed with a plasmid encoding a C-terminal polyhistidine tagged PbgA allele (PbgA^His-C^) or an empty plasmid. Like for the membranes, the LapB^Flag-C^ elution fractions from the whole-cell lysates from either growth phase contained both the chromosomal and plasmid-borne PbgA proteins (Fig. 8B). Chromosomal PbgA was again enriched in the LapB^Flag-C^ elution fractions for the stationary-phase *S*. Typhimurium relative to the log phase (Fig. 8A and B). Remarkably, unlike the LapB^Flag-C^ elution fractions from the membranes, the fractions from the whole-cell lysates of the stationary-phase bacteria contained multiple forms of LpxC including the 34 kDa predicted protein (Fig. 8B). The LapB^Flag-C^ elution fractions from log-phase *S*. Typhimurium also contained the 34 kDa form of LpxC but in reduced abundance (Fig. 8B). Further, PbgA overexpression did not influence the presence of LpxC in the elution fractions of the stationary-phase bacteria but routinely resulted in diminished LpxC co-elution in the LapB fractions of the log-phase bacteria (Fig. 8B). Therefore, *S*. Typhimurium differentially regulates the ability of LapB to bind LpxC as part of a constitutive PbgA-LapB complex that exists in both the log and stationary phase of bacterial growth.

### Like *E. coli*, PbgA interacts with LpxC in *S*. Typhimurium

**Fig 9:**
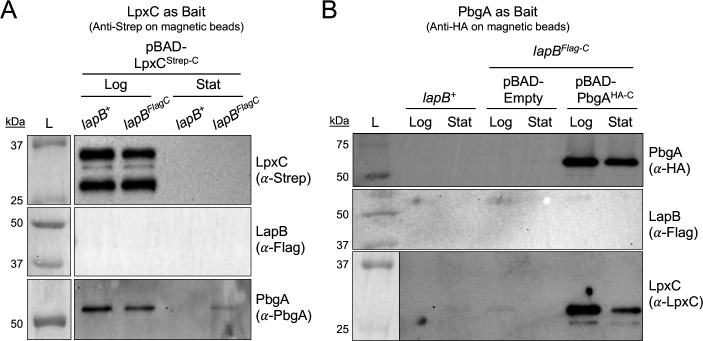
PbgA interacts with LpxC in *S*. Typhimurium during the logarithmic and stationary growth phase. (**A**) Immunoprecipitation of LpxC using magnetic beads loaded with anti-Strep antibodies from whole-cell lysates of 0.1% arabinose-supplemented cultures of *lapB^+^* and *lapB^Flag-C^* harboring a plasmid-borne copy of LpxC^∆293-305-Strep-C^ harvested in the logarithmic (Log) or stationary (Stat) growth phase. Equal amounts of bacterial lysates were incubated with the magnetic beads, washed, and then eluted. Fifteen microliters of the elution fractions was analyzed by SDS-PAGE and subjected to Western blotting for LapB (anti-Flag), LpxC (anti-Strep), and PbgA (anti-PbgA). The data are representative of three independent experiments. (**B**) Immunoprecipitation of PbgA using magnetic beads loaded with anti-HA antibodies from whole-cell lysates of 0.1% arabinose-supplemented cultures of *lapB^+^* or *lapB^Flag-C^* harboring a plasmid-borne copy of PbgA^HA-C^ or an empty vector harvested in the logarithmic (Log) or stationary (Stat) growth phase. Equal amounts of bacterial lysates were incubated with the magnetic beads, washed, and then eluted. Fifteen microliters of the elution fractions was analyzed by SDS-PAGE and subjected to Western blotting for LapB (anti-Flag), LpxC (anti-LpxC), and PbgA (anti-HA). The data are representative of three independent experiments. The lines tooled around the marker lanes are drawn to distinguish between two different image files of the same membrane. The left most image file of the ladder is the colorimetric image. In some instances, the secondary antibody interacts with the protein markers in the ladder; therefore, the chemiluminescent image alone cannot always be used.

The constitutive-complex model supports that PbgA could directly or indirectly associate with LpxC to control LpxC proteolysis. In fact, PbgA/YejM and LpxC interact in *E. coli* (28). To test whether PbgA binds LpxC in *S*. Typhimurium, we expressed LpxC^Δ293-305-Strep-C^ (LpxC^Strep-C^) from a plasmid in the *lapB^Flag-C^* genotype and immunoprecipitated LpxC^Strep-C^ from the whole-cell lysates of otherwise wild-type (*lapB^Flag-C^*) *S*. Typhimurium using anti-Strep antisera. Two predominant forms of LpxC^Strep-C^ migrating at roughly 36 and 30 kDa were routinely immunoprecipitated from the log-phase bacteria (Fig. 9A). Consistent with LapB and LpxC not showing an interaction during the log phase, LapB^Flag-C^ did not co-elute with LpxC^Strep-C^ in these cells (Fig. 8B and 9A). In contrast to LapB^Flag-C^, PbgA routinely co-eluted with LpxC^Strep-C^ under these conditions (Fig. 9A). Unfortunately, neither form of LpxC^Strep-C^ could be immunoprecipitated from the stationary-phase cells, due to the observed diminished levels (Fig. 2F and 9A). Given the inadequacy of the previous, PbgA was used as an alternative bait. To facilitate this approach, a C-terminal hemagglutinin tag was cloned on PbgA (PbgA^HA-C^) behind the regulatable promoter on the plasmid. The plasmid was introduced into the *lapB^Flag-C^* genotype, and the expression of PbgA^HA-C^ was induced. The anti-HA antibodies readily immunoprecipitated PbgA^HA-C^ from the lysates of both the log- and stationary-phase bacteria (Fig. 9B). Interestingly, the LpxC-specific antisera detected a smaller 30-kDA form of chromosomal LpxC in the elution fractions that contained PbgA^HA-C^ for both the log- and stationary-phase *S*. Typhimurium. The anti-Flag antisera did not detect LapB^Flag-C^ in the elution fractions from the log- and stationary-phase cells, suggesting that while LapB^Flag-C^ can precipitate PbgA, PbgA^HA-C^ cannot precipitate LapB^Flag-C^ under these conditions (Fig. 8). Attempts to detect the native LapB in the elutions using LapB-specific antibodies were also unsuccessful. The results strongly support that PbgA interacts with LpxC in *S*. Typhimurium. However, determining whether this interaction is physiologically or functionally relevant for regulating LpxC proteolysis will require future investigation.

## DISCUSSION

Our work provides a rigorous examination of LapB and its vital role in regulating LpxC levels and lipid A-core abundance in *S*. Typhimurium, a costly human pathogen. *S*. Typhimurium *lapB* mutants are viable but produce pinpoint-sized colonies on nutrient-rich agar, can hardly replicate in rich broth media, and are severely attenuated for colonizing mice during bacteremia (Fig. 2A and 3D; Fig. S1B). Consistent with *E. coli* work, our investigation highlights LapB as a major negative regulator of lipid A-core biosynthesis and enhancer of LpxC proteolysis. LapB activity is tightly regulated by PbgA, and LapB binds PbgA and LpxC (Fig. 6 to 8). Our study provides the first demonstration that LapB and PbgA interact in log- and stationary-phase bacteria and that the LapB-LpxC interaction is enriched in the stationary phase (Fig. 8). Therefore, contrary to the disassociation model, PbgA and LapB likely engage in constitutive interactions that modulate LpxC proteolysis in both log- and stationary-phase bacteria (Fig. 1C) (26, 28, 34, 36).

Similarly, Clairfeuille et al. concluded that PbgA-LapB could exist as a constitutive complex to regulate LpxC proteolysis, since the removal of the PbgA PD does not abrogate the PbgA-LapB interaction yet alters LpxC expression (33). If a constitutive complex exists in stationary-phase cells, then LapB must bridge PbgA and FtsH during proteolysis (Fig. 1C). If LapB brings PbgA in proximity to FtsH, then the PDs of PbgA and FtsH could intermittently engage in interactions as a tripartite TM heterocomplex that positively or negatively directs LpxC proteolysis (Fig. 1C) (35).

### How does the PbgA-LapB complex regulate LpxC proteolysis during the growth transition to stationary phase?

Diminished LpxC abundance in the LapB elution fractions from the log-phase bacteria relative to the stationary phase suggests that when nutrients are replete, LapB is structurally or conformationally inhibited from binding to LpxC (Fig. 8A and B). Consistent with this hypothesis, PbgA overexpression diminished LpxC abundance in the LapB elution fractions for the log phase but not the stationary-phase bacteria (Fig. 8B). Therefore, PbgA-LapB must exist in LpxC-bound and unbound states.

Adding further complexity to the already sophisticated signal transduction system, PbgA interacts with LpxC in log- and stationary-phase bacteria (Fig. 9). The requirement of LapB for this interaction or its impact on LpxC proteolysis remains uncertain. Nonetheless, it is possible that PbgA binds to LpxC, preventing LapB from binding until LPS signals are received at the periplasm-IM interface. According to this reasoning, LpxC transfer mechanisms between PbgA and LapB at the cytosol-IM interface could govern LpxC proteolysis, which would support the constitutive-complex model (Fig. 1C). By this rationale, PbgA-LPS binding in the periplasm could induce PbgA to transfer LpxC to LapB in the cytosol; however, further biochemical work is required. A major caveat here is that we were unable to capture LapB when PbgA was used as bait. This could either be a physiological phenomenon or simply a consequence of the position of the epitope tag on PbgA, which might restrict its ability to interact with LapB under certain conditions.

### Does *S*. Typhimurium utilize LapB to regulate proteolysis of additional cell envelope biosynthesis enzymes?

Work in *E. coli* highlights LapB as a promiscuous binding protein that interacts with other key enzymes for lipid A-core and GPL biosynthesis including LpxA, LpxD, and FabZ. Like LpxC, LapB also promotes LpxD proteolysis (28). Therefore, Enterobacteriaceae likely use PbgA-LapB to regulate multiple FtsH substrates (52, 53). LapB interactors might occupy the same or different binding sites as LpxC as part of regulated proteolysis (51). Deciphering critical LapB regions and residues for these and other interactions including with metals like Zn^2+^ and Fe^2+^ will allow a better understanding of LapB mechanisms necessary for enterobacterial cell envelope biosynthesis and regulation (24, 47).

Substitutions in LpxA and LpxC restore bacterial growth and LPS regulation for *lapB* deletion mutants but do not restore LpxC expression levels, suggesting that the mutations perturb another mechanism (Fig. S2; Table S3). The residues are positioned near the homotrimeric interface and catalytic sites of LpxA and LpxC, respectively, suggesting possible conformation obstruction or catalytic perturbation as suppressive mechanisms (Fig. S5) (54, 55). These mutations likely cause decreases in LpxA and LpxC activity that reduce substrate usage by the lipid A biosynthesis pathway (Fig. S2). By this reasoning, LapB’s key function is to reduce or limit the rate of lipid A-core biosynthesis in these bacteria.

### What is the structure of the LapB-LpxC complex *in vivo*?

We have interrogated an LapB-LpxC model and concluded that LapB’s cytoplasmic domain is sufficient for LpxC binding (Fig. 6 and 7). Top-ranked LapB monomer and homodimer models positioned LpxC near TPRs 2–4 (Fig. 7A; Fig. S4B). Using a co-expression and purification approach, we established that the LapB-LpxC interaction involves residues within TPRs 2–4 (Fig. 7B through E). We provide evidence to support that LapB’s TM segment, TPR 1, TPRs 5–9, and the rubredoxin as well as LpxC’s C-terminal extension are not necessary for LapB-LpxC binding under these conditions (Fig. 6 and 7). However, the exact contacts between LapB and LpxC necessary for LapB function toward promoting LpxC proteolysis and lipid A-core downregulation in *S*. Typhimurium remain undetermined.

The functional oligomeric state of LapB is not understood; however, previous work has shown that the polypeptide forms homodimers, while in this study, homotrimers were observed (27, 47). Although homoligomerization of TPR-containing proteins is common, our results do not speak as to whether LapB oligomerization is necessary for LpxC binding or influences PbgA or FtsH binding (56). Crystallizations support that C-terminal TPRs mediate homodimerization, and the N-terminal residues through TPR 3 are not required (27, 47). The concave surface formed by TPRs 8–9 elaborates a hydrophobic tunnel predicted to interact with lipid molecules, which could signal multimerization (27). It will be critical to understand whether controlling LapB oligomerization is a mechanism for bacteria to regulate LapB function.

Cryo-EM interrogation of FtsH reported flexibility within the proteolytic chamber near the cytosol-IM interface, and this might be a determinant for substrate entry (57). Interrogation of the conformation of *S*. Typhimurium LapB versus *E. coli* LapB denoted a significant tilt in one protomer of the *S*. Typhimurium LapB homodimer (27). Given the proximity of LapB TPRs 2–4 to the cytosol-IM interface, this could be a mechanism to localize LpxC near FtsH’s proteolytic site to enhance LpxC digestion. Rotation or tilting could alter the LapB TPR binding mechanisms by impacting the position of the concave interface. By this reasoning, periplasmic interactions between PbgA and LPS molecules could emit a signal through the TMs of PbgA-LapB resulting in rotation or tilting of the LapB multimer to optimally position the concave interface for LpxC binding. Deleting the helical region preceding TPR1 and TPR1 itself did not impact LpxC co-elution (Fig. 7C). Therefore, the short helical extension between the TM and TPR1 might interact with the proteolytic chamber or ATPase region of FtsH as part of enhancing proteolysis (Fig. S3).

We have discussed our interpretations of the function and mechanisms of LapB for *S*. Typhimurium from the perspective of LapB as a component of a multiprotein complex that controls cell growth and survival of Enterobacterales by regulating LPS biogenesis. This critical regulatory system works by responding to the environment through protein–glycolipid and protein–protein binding mechanisms and directed proteolysis of a rate-limiting target for pre-clinical antibiotics, LpxC, a tactic that enables bacteria to control LPS abundance on their surface.

## MATERIALS AND METHODS

### Bacterial strains and culturing conditions

All bacterial strains and plasmids used in this study are listed in Table S1. Culturing was routinely performed in LB medium (0.1% tryptone, 0.5% yeast extract, and 0.5% NaCl) supplemented with antibiotics and sugar additives where indicated. *E. coli* DH5α was cultured at 37°C and used as a host for plasmid recombination and cloning. *E. coli* BL21 (DE3) was cultured at 30°C and 37°C and used as a host for protein overproduction and purification. Plasmid selection and maintenance for pBAD24 required 100 µg/mL ampicillin and 10 µg/mL chloramphenicol for pACYC-Duet1. The bacterial strains used for the *in vivo* transcomplementation and immunoprecipitation studies were derivatives of the *S*. Typhimurium 14028s, which contains a phenotypically neutral and chromosomally integrated *wza-lacZ* gene promoter fusion (31, 32, 37).

Each strain was streaked onto LB agar plates containing the LacZ indicator substrate, 5-bromo-4-chloro-3-indolyl β-D-galactopyranoside (X-Gal) (VWR) at a concentration of 20 µg/mL with antibiotics when necessary. The bacteria were isolated from −80°C glycerol stocks weekly. Cultures were routinely started with either a single colony inoculated into LB medium and shaken or rotated at 250 rpm aerobically at 30°C, 37°C, and 42°C or 1:100 back-diluted into fresh LB. L-Arabinose (Sigma) at 0.1% vol/vol was used for *in vivo* overexpression, 0.2% vol/vol glucose (Sigma) for plasmid repression, and 1 mM isopropyl β-d-1-thiogalactopyranoside (IPTG) (Sigma) for protein overexpression and purification. The logarithmic growth phase was defined as an optical density at 600 nm (OD_600_) of 0.6–0.8, and the stationary growth phase was established as the culture density achieved after a 16-h post-single-colony inoculation at indicated temperatures.

### Bacterial genetics

All primers used in this study are provided in Table S2. Chromosomal epitope tagging of *lapB* was achieved using the system described by Führer et al. (48) (Table S1). All deletion–insertion mutants were created using a phage lambda red recombinase system, and P22 bacteriophage transduction was used to move *lapB::tetRA* alleles between *S*. Typhimurium genotypes (58).

The pACYC-Duet1 plasmid and Gibson assembly were used to recombinantly modify LapB and LpxC for protein overexpression and purification studies of mutagenesis to assess the role of key LapB regions and residues for binding. LapB TPR motifs (~34 amino acids, 102 bp) were designated according to AlphaFold2 predicting *S*. Typhimurium LapB-LpxC models and previous published LapB studies (49). Individual LapB TPRs were sequentially deleted with direction from structural analysis and visualization using ChimeraX. Glycine residues within the looping regions between two adjacently stacked TPRs were targeted. This began with the removal of the LapB transmembrane (LapB^∆TM^ = LapB^∆1-22aa^) and subsequent TPRs including LapB^∆TM-TPR1^ (LapB^∆1-67aa^), LapB^∆TM-TPR2^ (LapB^∆1-96aa^), and LapB^∆TM,TPR5-Rdx^ (LapB^∆1-22aa,214-389aa^).

### Quantifying protein concentration

Protein concentrations were measured using the Pierce Coomassie Plus Bradford assay reagent (Thermo Scientific Cat. # 23238) and a standard curve generated from bovine serum albumin standard.

### Bacterial growth curves

A growth curve was generated by the back-dilution (1:100) of stationary-phase cultures into 250 mL of LB supplemented with ampicillin (100 µg/mL) and 0.1% arabinose addition to culture media after reaching the logarithmic phase. Bacteria were incubated with continuous agitation, and OD_600_ was measured and recorded once per hour (*n* = 3, ±SEM).

### β-Galactosidase assays

β-Gal assays were performed using standard procedures. Briefly, 5–10 mL of log- or stationary-phase bacterial cultures were pelleted and resuspended in Z-buffer (0.06 M Na_2_HPO_4_, 0.04 M NaH_2_PO_4_, 0.01 M KCl, and 0.001 M MgSO_4_ 7H_2_O). OD_600_ readings were recorded to quantify culture density. Bacteria were permeabilized with chloroform and 0.1% (wt/vol) SDS. The time to develop a yellow color was assessed following the addition of ortho-nitrophenyl-b-galactosidase (4 mg/mL in PBS) and incubation at 30°C. 1 M Na_2_CO_3_ was added to stop the reaction, and the mixture was centrifuged to remove any contaminants. The supernatant was withdrawn and transferred to sterile cuvettes, and optical density readings at 420 nm (OD_420_) were analyzed. The levels of LacZ or β-Gal activity was calculated according to the formula (OD_420_)/(time × volume × OD_600_) (*n* = 3, ±SEM).

### Harvesting whole-cell lysates and isolating total membranes

One-liter cultures were collected at specific growth phases and centrifuged at 8,000 rpm. The pellets were resuspended in 10 mL of PBS and incubated for 30 min at 4°C with lysozyme (144 µg/mL) (VWR Cat. # 97062-136), 1 µL of protease inhibitor cocktail EDTA-free (100×) (Thermo Cat. # 78425), and 1 µL of RNAse/DNAse nuclease reagent (Sigma Cat. # 70746-3). The treated *S*. Typhimurium bacteria were homogenized and lysed using Emulsiflex-C3 (Avestin) until the sample was clear. After centrifugation at 8,000 rpm, the whole-cell lysates were ready to use or freeze. If total membrane harvesting was desired, the previous lysate was ultracentrifuged at 40,000 rpm for at least 2 h at 4°C. Membranes were resuspended in 1 mL of PBS.

### Western blotting

Cell pellets from 5–10 mL cultures were collected and resuspended in 175 µL of PBS and 25 µL of 4 × Laemmli buffer (Bio-Rad Cat. # 1610747). Samples were boiled for 10 min in a water bath and incubated on ice for 2 min. Similarly, when blotting using total membrane samples, 40 µg of total proteins/sample was mixed with Laemmli buffer (Bio-Rad Cat. # 1610747), boiled for 2 min, and sonicated. Fifteen microliters of the supernatants was loaded onto a 10% SDS-PAGE gel, electrophoresed, and transferred onto a polyvinylidene fluoride membrane (Fisher Cat. # 45-004-021) using the Mini Trans-Blot Cell (BioRad) apparatus for wet transfer at 100 V for 120 min at 4°C. The membrane was washed in Tris-buffered saline supplemented with 1% vol/vol Tween 20 (TBS-T) and blocked overnight at 4°C in 5% non-fat dried milk/TBS-T. For chromosomal LapB^Flag-C^, the monoclonal FLAG tag antibody (BioLegend, Clone L5, Cat. # 637304) was diluted 1:1,000 in TBS-T and incubated with the membrane at 4°C overnight. For the plasmid-borne LapB^6xHisC^ and LapB^∆TM6xHisC^, a primary monoclonal α-His antibody (ProteinTech Cat. # 66005-1-Ig) was diluted 1:1,000 in TBS-T overnight at 4°C. To blot chromosomal LpxC and DnaK, polyclonal antibodies to LpxC (MyBioSource Cat. # MBS1488471), DnaK (MyBioSource Cat. # MBS565041) were diluted 1:10,000 in TBS-T and incubated at room temperature for 4 h. Anti-PbgA^191-586^ antibodies were obtained previously and cleared from rabbit-antisera as described (37). To blot PbgA, a dilution of 1:250 in TBS-T was used and incubated overnight at 4°C. For the plasmid-borne LpxC^Strep-N^ blots, the primary α-Strep monoclonal antibody (G-Bioscience Cat. # ITM3016) was diluted 1:1,000 in TBS-T overnight at 4°C. As secondary antibodies, α-rat IgG, HRP-linked antibody (Cell Signaling Cat. # 7077S), α-mouse IgG, HRP-linked antibody (Cell Signaling Cat. # 7076S), and α-rabbit IgG, HRP-linked antibody (Cell Signaling Cat. # 7074S) were diluted 1:10,000 in TBS-T and incubated accordingly with the membranes for 45 min at room temperature. Blots were imaged after detection with the SuperSignal West Pico Plus detection reagent (Thermo Scientific Cat. # 34577) using a BioRad ChemiDoc MP Imager.

### Extracting and visualizing LPS

The method for extracting, visualizing, and quantifying LPS molecules was slightly modified from previous publications (32). A single colony of each bacterial genotype was inoculated into 8 mL of LB and incubated aerobically at the indicated temperatures (30°C, 37°C, or 42°C) overnight for 16 h. Liquid cell cultures were normalized to an OD_600_ of 2.5, and the required volume was aliquoted into a 2-mL microcentrifuge tube. The cells were pelleted by centrifugation at 14,000 rpm for 5 min. The supernatants were carefully discarded using a pipette, and the pellets were resuspended in 200 µL of sterile water by vigorous pipetting without vortexing. A 20-µL aliquot of the resuspended cells was reserved, serially diluted, plated onto LB agar, and enumerated to ensure that the extracted LPS was obtained from similar numbers of bacteria across various genotypes. The remaining cell suspension (180 µL) was denatured via SDS/boiling lysis for 10 min, removed from heat, and reequilibrated to room temperature. Once cooled, samples were treated with 5 µL of Proteinase K solution (20 mg/mL) (NEB) and incubated in a 59°C water bath overnight (~16–18 h). Utilizing a hot phenol water extraction, 180 µL of prewarmed (68°C) Tris-saturated phenol (pH 8.0) was added to each sample and vortexed. Samples were incubated in a 68°C water bath for 15 min and immediately transferred to an ice-cold water bath for 10 min. To achieve proper phase separation, samples were centrifuged at 4,000 rpm for 10 min at 4°C. The upper aqueous phase containing the LPS molecules (~150 µL) was carefully extracted using a pipette and placed in a sterile microcentrifuge tube. For SDS-PAGE, 30 µL of lipid samples was diluted into 10 µL of 4 × Laemmli buffer (Bio-Rad). The LPS-containing extracts (20 µL per well) were separated on a 4%–20% Tris-glycine gradient gel (Bio-Rad) and electrophoresed at 100 V for 45 min. Gels were stained according to the Pro-Q Emerald 300 LPS Gel Stain Kit (Molecular Probes), and the relative fold change in band intensity was quantified using BioRad Image Lab 6.0.1 software.

### Infecting mice

Male and female C57BL/6J mice were purchased from The Jackson Laboratory and bred in-house under pathogen-free conditions. To measure the ability of *S*. Typhimurium to survive systemically and colonize the spleens and livers of mice, 6- to 8-week-old animals were intraperitoneally injected with 5 × 10^5^ cfu diluted in PBS. At 48 h, the mice were euthanized, and the livers and spleens were dissected, weighed, and homogenized in PBS-0.1% Triton X-100. The organ homogenates were serially diluted and plated onto LB agar + amp + X-Gal to enumerate colony-forming units.

### Antibiotic sensitivity

Plating efficiency on rifampin (Rif) was determined by adjusting the density of 5 mL log-phase cultures to OD_600_ of 1.0 after 2 h of incubation with 0.1% arabinose added to culture media. Cells were serially diluted and spot-plated onto LB agar supplemented with X-Gal, with or without Rif at 2.5 µg/mL. The plates were incubated at 37°C overnight for 16 h.

### Immunoprecipitation assays

For LapB^Flag-C^, PbgA^6-His-C^, or LpxC^Δ293-305-Strepp-C^, Pierce Protein-G magnetic beads (Thermo Scientific Cat. # 88847) were loaded with α-FLAG antibodies (BioLegend, Clone L5, Cat. # 637304), α-6xHis monoclonal antibodies (ProteinTech, Cat. # 66005-1-Ig). Each immunoprecipitation reaction consisted of 25 µL of washed beads, resuspended in 10 µg of antibody/100 µL of modified coupling buffer, consisting of a (1.9:0.1) mix of Buffer A (115 mM NaCl, 1.2 mM CaCl_2_, 1.2 mM MgCl_2_, 2.4 mM K_2_HPO_4_, and 20 mM HEPES) and IP lysis/washing buffer (Tris Base 25 mM, NaCl 150 mM, EDTA 1 mM, NP40 1%, and glycerol 5%), following Pierce Crosslink Magnetic IP/Co-IP Kit instructions. The beads and antibodies were incubated on a rotating platform for 15 min at room temperature (~20°C). The antibodies were covalently attached to the magnetic beads by adding a disuccinimidyl suberate (OneQuant DSS) (G-Biosciences Cat. # BC04-Q) cross-linking reagent at a final concentration of 20 µM and incubating at room temperature on a rotating platform. Glycine buffer (0.1 M, pH 2.5) was used to quench the reaction and wash in excess. For PbgA^HA-C^, Pierce Anti-HA magnetic beads (Thermo Scientific Cat. # 88836) were washed adequately in 0.05% TBS-T (Tris-buffered saline containing 0.05% Tween-20 detergent). Total protein (4 mg) from the membrane fractions or whole-cell lysates (10 mg) of bacteria cultured to the log or stationary phase of growth was diluted in 0.5 mL of 1% n-dodecyl-beta-D-maltoside (Adipogen Cat. # 102987-834) in water and incubated at 4°C for 4 h on a rocking platform. The solubilized membranes were then added to the loaded beads and incubated at 4°C overnight on a rocking platform. The beads were washed three times with the IP lysis/washing buffer or TBS-T (for the anti-HA beads). In all cases, the proteins bound to the beads were eluted with glycine buffer (0.1 M, pH 2.5). Fifteen microliters of elution fractions was loaded onto and separated by 10% SDS-PAGE, transferred, and blotted against α-Flag (1:1,000), α-PbgA (1:250), α-His (1:10,000), α-Strep (Immunotag Strep-tag monoclonal Ab) (Clone 7A10) (G-Biosciences Cat. # ITM3016) (1:5,000), α-LpxC (MyBioSource Cat. # MBS1488471) (1:10,000), α-DnaK (MyBioSource MBS565041) (1:10,000), or α-HA (Roche, Clone F90, Cat. #11867423009) (1:10,000).

### Purifying ^6xHisN-^LapB^ΔTM^

Stationary-phase cultures of *E. coli* BL21 harboring the expression plasmid pACYC1-^6xHisN-^LapB^ΔTM^ were back-diluted 1:100 into 1 L of LB, grown at 250 rpm at 37°C until an OD_600_ of 0.5, and induced by adding 1 mM IPTG to culture media. Induced cultures were grown at 30°C for an additional 4 h. Cultures were pelleted by centrifugation at 8,000 rpm for 10 min and resuspended in Buffer M (10 mM MES and 150 mM NaCl at pH 6.5) supplemented with lysozyme (1 mg/mL), benzonase nuclease (3 U/mL of lysate) (Millipore Sigma Cat. 70746-3), and protease inhibitor (ProteCEASE-50, G-Biosciences Cat. 786-326). The cell resuspension was shaken vigorously on a rocking platform at 4°C for 30 min. Cells were lysed under high pressure (20,000 psi) using an Emulsiflex-C3 (Avestin), centrifuged at high speed, and further separated into a soluble and membrane fraction by ultracentrifugation at 40,000 rpm for 1 h. The soluble fraction was recirculated for 1–2 h over a 5 mL HisPur cobalt resin column (Thermo Scientific) pre-equilibrated with an excess of Buffer M. The protein-charged column was then attached to an AKTA Prime Plus FPLC system (Cytiva). ^6xHisN-^LapB^ΔTM^ was purified by washing the cobalt column with 20 mL of Buffer M and eluted by pumping in a linear gradient (0%–100%) of Buffer M2 (10 mM MES, 150 mM NaCl, and 1 M imidazole, pH 6.5). Peak fractions were identified using AKTAPrime software, collected, and verified for purity by SDS-PAGE and Western blotting. Verified fractions were concentrated using a 5–20 mL 10 K MWCO (Pierce Protein Concentrator, PES Cat. # 88528) as per the manufacturer’s protocol. A second step of affinity purification using size exclusion chromatography and a Superdex 75 column was used to generate pure ^6xHisN-^LapB^ΔTM^ as judged by SDS-PAGE compared to the elution fraction post-cobalt purification. Pure fractions were pooled and dialyzed into HEPES buffer containing 20 mM HEPES, 300 mM NaCl, and 25% glycerol, pH 7.0, and aliquoted to a final 1 mg/mL concentration and stored at −20°C. The same upscaled purification conditions were used to purify the co-overexpressed ^6xHisN-^LapB^ΔTM^-LpxC^Δ293-305-S-tag^.

### Microscale co-purification using the dual expression vector

Overnight cultures of *E. coli* harboring the dual expression plasmid pACYC1-^6xHisN^LapB^Δ1TM^-LpxC^Δ294-305-S-tag^ were back-diluted 1:100 into 1 L of LB and cultured at 37°C shaking at 250 rpm until reaching an OD_600_ of 0.5. Cultures were processed as described previously, except using an alternate buffer system consisting of a wash/lysis buffer NPI-10 (50 mM sodium phosphate, 300 mM NaCl, and 10 mM imidazole, pH 7.0) and an elution buffer NPI-200 (50 mM Na_2_HPO_4_, 300 mM NaCl, and 200 mM imidazole, pH 7.0). The Dynabeads His-Tag Isolation and Pull-down magnetic cobalt bead system (Invitrogen Cat. #10103D) was utilized to capture ^6xHisN^LapB^Δ1-22^ (bait) to determine if the co-expressed protein of interest LpxC^Δ293-305^-S-tag (prey) co-eluted. The following procedure was used for all purifications in this analysis: 50 µL of magnetic cobalt beads was washed and equilibrated with 2 mL buffer NPI-10 in a 2-mL microcentrifuge tube and then resuspended in 2 mL of soluble lysate normalized to a total protein concentration of 2.5 mg/mL and rocked on a platform shaker for 16 h at 4°C. Beads were washed with 2 mL of a stepwise gradient of buffer NPI-10 supplemented with increasing imidazole concentrations (20–60 mM). Captured ^6xHisN^LapB^Δ1-22^ was eluted by the addition of 200 µL of Buffer NPI-200 rocking at 4°C for 30 min. The protein concentration in isolated fractions was estimated using the Coomassie Plus Bradford Assay (Thermo Fisher Cat. # 23236), and a volume equal to 2.5 µg was resuspended in 10 µL of 1× Laemmlli buffer, loaded onto a 10% SDS-PAGE gel in duplicate to verify purity by Coomassie staining and protein presence via immunoblotting. The presence or absence of bait and prey proteins in eluted fractions was validated by utilizing tag-specific antibodies α-6xHis (ProteinTech Cat. # 66005-1-Ig) and α-S-Tag (Novus Biological Cat. # NB600-511). Similar purifications were performed for single expressed protein controls for pACYC-^6xHisN-^LapB^Δ1-22^_EMPTY to establish protein size, pACYC-EMPTY_LpxC^Δ294-305^-S-tag as a non-specific binding control.

### Native gel electrophoresis

The single buffer system, 25 mM Tris, 192 mM glycine, pH 9.0, was used to separate 5 µg of purified ^6xHisN^LapB^∆TM^ or co-purified ^6xHisN^LapB^∆TM^-LpxC^∆293-305-S-Tag^ equally loaded onto 4%–20% gradient gels (BioRad Cat. # 4568095) and run at 100 V for 2.5 h at 4°C. Gels were rinsed three times with sterile water and Coomassie-stained for visualization using a ChemiDoc MP with standard settings.

### Gel filtration chromatography

^6xHisN^LapB^∆TM^ or ^6xHisN^LapB^∆TM^-LpxC^∆293-305-S-Tag^ samples post-cobalt purification were applied to gel filtration chromatography on a Superdex 75 Increase 10/300 GL column (Cytiva Cat. # 29148721) for a second step of affinity purification using buffer containing 25 mM Tris and 300 mM NaCl pH 7.5 at a flow rate of 1 mL/min. Peak fractions encompassing the single peak were concentrated and judged by comparison to the cobalt elution fractions on a Coomassie-stained 10% SDS-PAGE gel for purity followed by analysis via native gel electrophoresis (Fig. 6D and E). Pure fractions were pooled and dialyzed into HEPES buffer containing 20 mm HEPES, 300 mM NaCl, and 25% glycerol, pH 7.0, and aliquoted to a final concentration of 1 mg/mL and stored at −20°C.

For molecular weight estimation, cryo-preserved LapB and LapB-LpxC samples were diluted to a final concentration of 0.5 mg/mL in HEPES storage buffer with a reduced glycerol content (10%) and supplemented with 1 mM dithiothreitol (DTT) prior to loading. Samples were passed through a 0.8-µm filter and loaded onto a Superdex 200 Increase 10/300 GL column (Cytiva Cat. # 28990944) pre-equilibrated with sample buffer. Prepared LapB and LapB-LpxC samples were injected and captured in 1 mL fractions at a flow rate of 0.5 mL/min. Confirmation of protein presence was accomplished by analyzing fractions encompassing the isolated peak by mixing 15 µL of samples with 5 µL of 1× Laemmlli buffer followed by loading, separation, and Coomassie staining on a 10% SDS-PAGE gel. A protein standard consisting of bovine thyroglobulin (670 kDa), bovine gamma-globulin (158 kDa), ovalbumin (44 kDa), myoglobulin (17 kDa), and vitamin B12 (1.35 kDa) was utilized to generate a standard curve and estimate the molecular weight of LapB or LapB-LpxC based on the peak elution timepoint (Fig. 6C).

### Graphical illustrations

Graphical illustrations were created with BioRender.com

### Structural modeling

AlphaFold2 was used via ColabFold to predict the structures of *S*. Typhimurium LapB, LpxC, and LapB-LpxC complexes (59, 60). Corresponding protein sequences were submitted individually and together to determine the likelihood of interaction and potential region of interaction. All structural models analyzed and figures within the publication were prepared using UCSF Chimera (61). The top-ranking model used for this study was selected based on visual inspection, ipTM+pTM, and pLDDT scores (Table S4). LpxC suppressor mutations were mapped by utilizing the Chimera matchmaker function comparing AlphaFold2 generated *S*. Typhimurium LpxC to *E. coli* LpxC crystallized with its native substrate (PDB code: 4ZLH) (57). This method was also used to map LpxA suppressor mutations to the published *E. coli* LpxA biological assembly structure (PDB code: 2QIA) (68).

### Statistical analysis

All statistical analyses were performed, and graphs were prepared using GraphPad Prism (version 8; GraphPad Software, La Jolla, CA, USA).

## SUPPLEMENTAL MATERIAL

Supplemental material jb.00308-23-s0001.docx Fig. S1 to S5 and Tables S1 to S4.

ASM does not own the copyrights to Supplemental Material that may be linked to, or accessed through, an article. The authors have granted ASM a non-exclusive, world-wide license to publish the Supplemental Material files. Please contact the corresponding author directly for reuse.
