# Supplementary material for: Signaling through the Salmonella PbgA-LapB regulatory complex activates LpxC proteolysis and limits lipopolysaccharide biogenesis during stationary-phase growth
Source: J Bacteriol. 2024 Mar 27;206(4):e00308-23. doi: 10.1128/jb.00308-23 (PMC11025326; doi:10.1128/jb.00308-23)
Supplement: Supplemental material — Fig. S1 to S5 and Tables S1 to S4. [file jb.00308-23-s0001.docx]

**Supplementary Information for:**

**Signaling through the *Salmonella* PbgA-LapB regulatory complex activates LpxC proteolysis and limits lipopolysaccharide biogenesis during stationary phase growth.**

**AUTHORS:** Joshua A. Mettlach^1^, Melina B. Cian^1^, Medha Chakraborty^1^, and Zachary D. Dalebroux^1^*

**AFFILIATIONS:** ^1^Department of Microbiology and Immunology, University of Oklahoma Health Sciences Center, 940 Stanton L. Young Blvd., BMSB 1053, Oklahoma City, OK 73104, USA.

***Correspondence to:**

Zachary David Dalebroux, Ph.D.

734-255-9330

Zachary-Dalebroux@ouhsc.edu

**Supplementary Figure 1.**


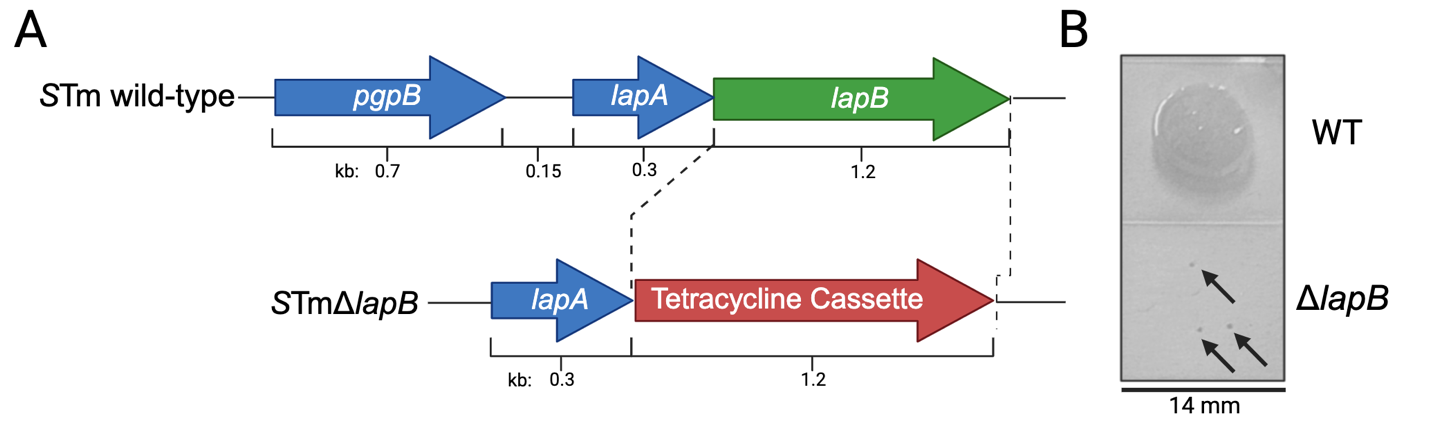
**Figure S1. *S.*Typhimurium *lapB* deletion results in pinpoint sized colonies on nutrient-rich agar media.** (**A**) Graphical illustration of the *lapAB* operon, promoters, and relative gene size (upper panel) (1, 2). Schematic of the *lapAB* operon with sequence-validated chromosomally integrated tetracycline cassette in place of *lapB* (lower panel). (**B**) A single colony of wild-type (*lapB^+^)* and *∆lapB* mutant was resuspended in 50µl of LB and 2µl was spot-plated onto LB agar supplemented with 20μg/ml β-galactosidase (LacZ) indicator substrate X-Gal (5-bromo-4-chloro-3-indolyl-β-d-galactopyranoside) and cultured at 30°C for 16h. Arrows indicate ∆*lapB* mutants colony size in comparison to WT.

**Supplementary Figure 2**

**
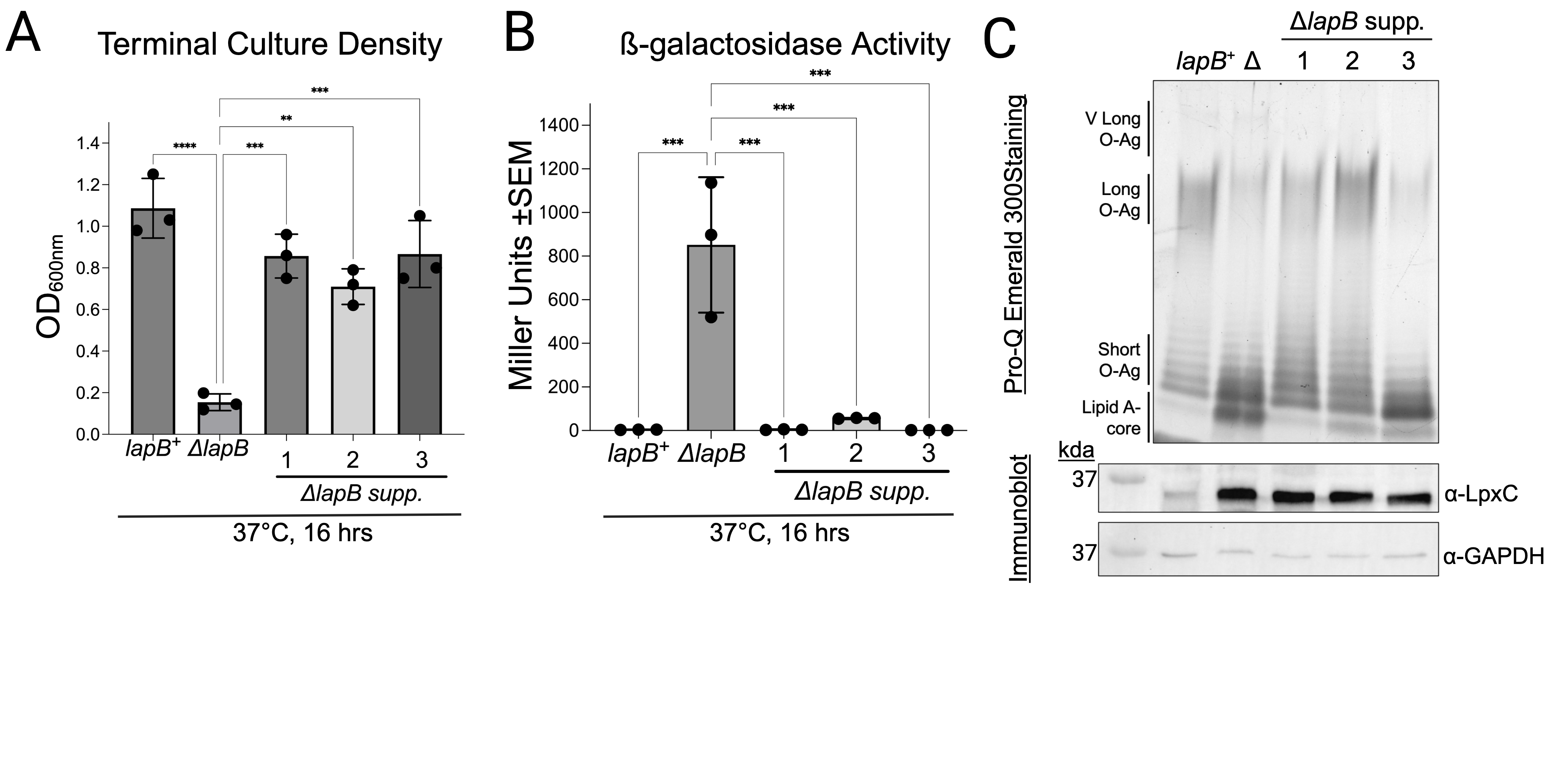
Figure S2.** ∆*l****apB* mutant suppressor isolates restore growth and OM integrity defects irrespective of aberrant LpxC accumulation** (**Table S3**). (**A**) Terminal growth culture density and (**B**) ß-galactosidase activity were measured for wild-type (*lapB^+^)* *∆lapB* mutant, and ∆*lapB* mutant suppressor isolates cultured at 37°C for 16 hr. Statistical significance was calculated using one way-ANOVA followed by Tukey’s multiple comparison test comparing the mean of each column with the mean of every column (*,p<0.0332, **, p<0.0021, ***,p<0.0002, ****, p<0.0001) (n=3, ± SEM). (**C**) Analysis of LPS and LpxC levels of wild-type (*lapB+)* *∆lapB* mutant, and ∆*lapB* mutant suppressor isolates cultured at 37°C for 16 hr. LPS samples were equivalently loaded (15 µl), separated using a 4-20% SDS-PAGE gel, and visualized by Pro-Q 300 Emerald staining. For immunoblotting, cells were normalized to an OD_600_ of 2.5 after 16 hr of culturing, resuspended in a 4:1 mixture of PBS:Laemmli buffer, subjected to SDS-boiling lysis, equivalently loaded (15 µl) and separated using a 10% SDS-PAGE gel. α-GAPDH was used as a loading control. Data are representative of three biological replicates.

**Supplementary Figure 3.**

**
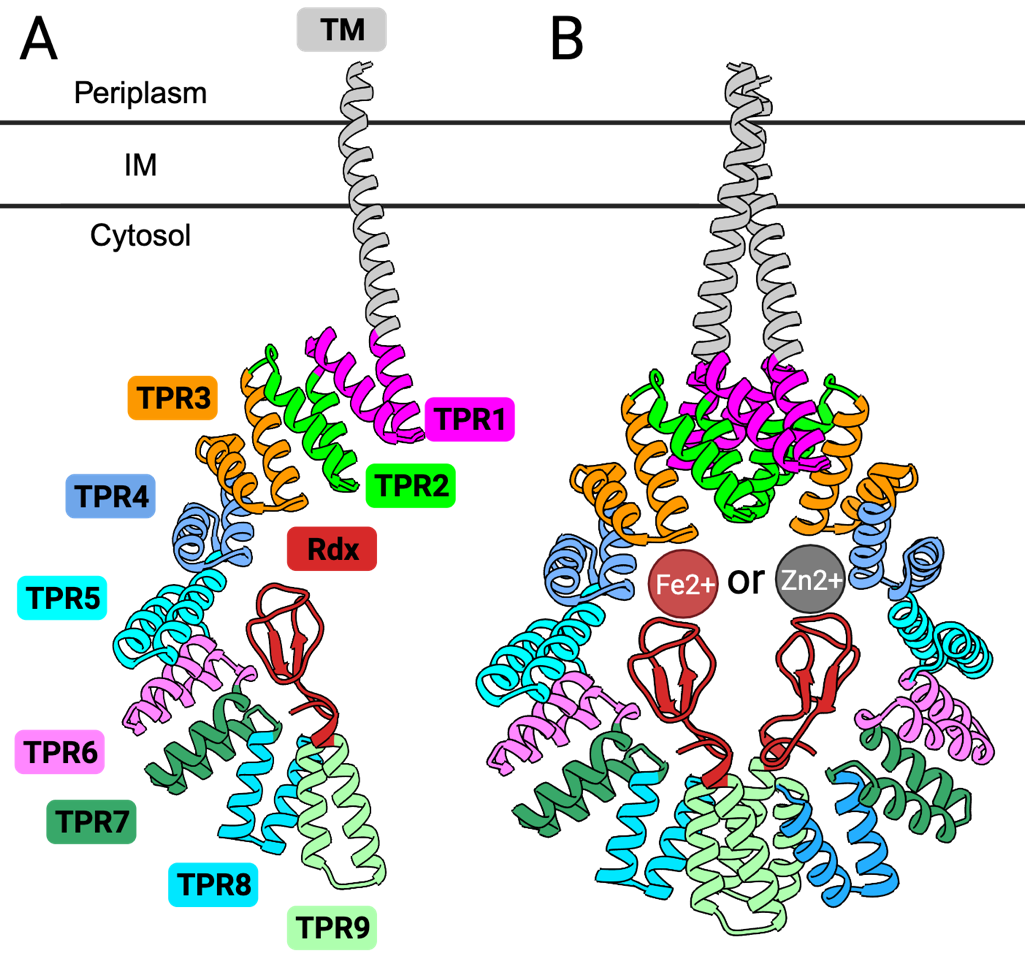
**

**Figure S3.** **LapB Structure.** AlphaFold2 predicted model of the (**A**) LapB monomer and (**B**) homodimer, which has been variably colored to emphasize and distinguish the positioning and shape imparted by the nine consecutive TPRs. The LapB C-terminus carries a metal-binding Rdx domain that binds Zn^2+^ or Fe^2+^(3-6).

**Supplementary Figure 4.**

**
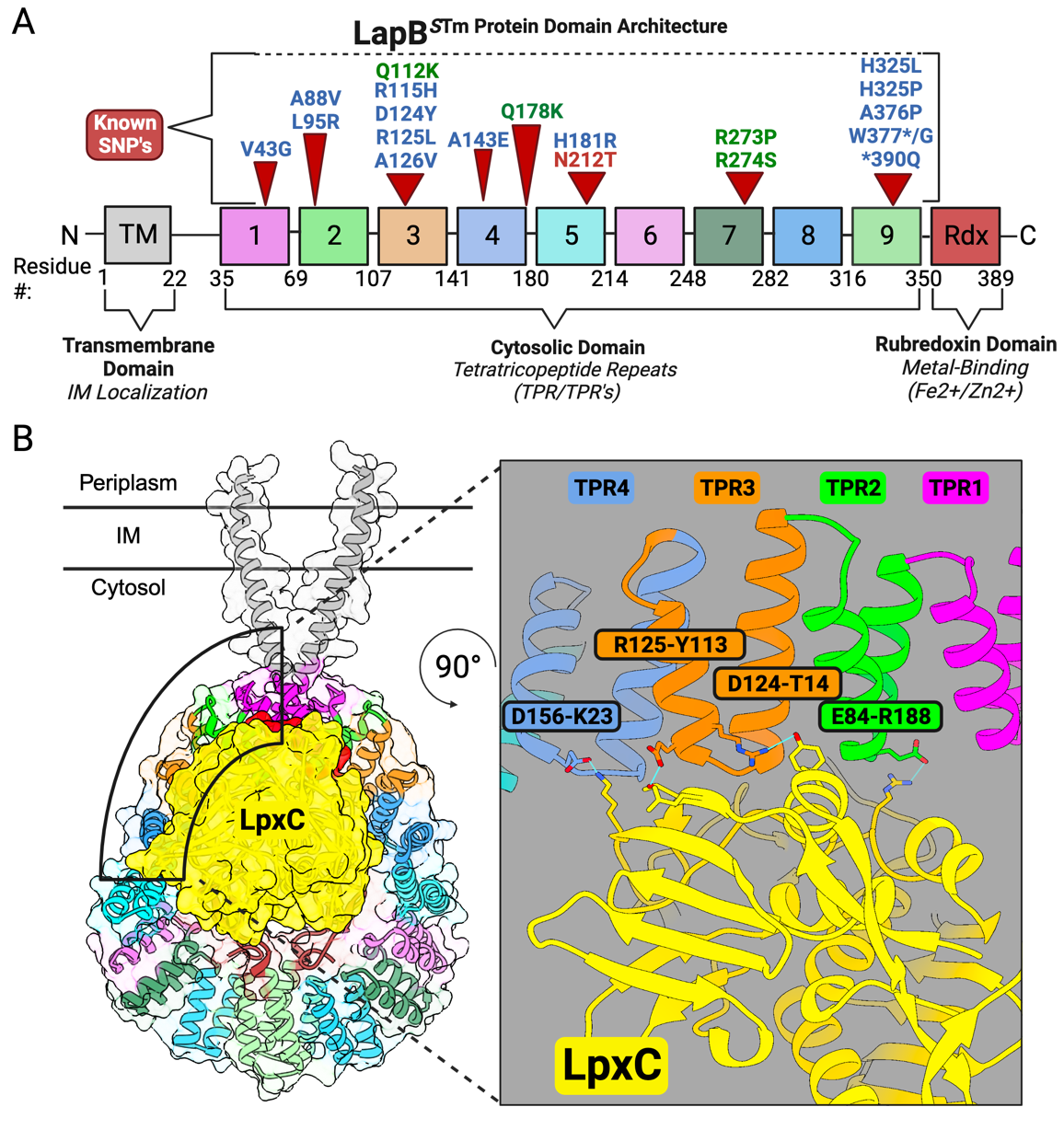
**

**Figure S4.** **Biologically relevant SNPs in LapB are positioned with the AlphaFold2 predicted LapB-LpxC interaction interface.** (**A**) Linear schematic of the domain architecture of LapB and the location of single nucleotide polymorphisms (SNPs) in *lapB* that have been isolated as suppressors of defects in LpxC and LPS regulation and polymyxin sensitivity. Text in green signifies *lapB* SNPs isolated in our studies (7). Blue text refers to *lapB* SNPs and site-directed substitutions reported in *E. coli* studies (8-12). Red text refers to a *lapB* SNPs isolated in *Klebsiella pneumoniae* (13). (**B**) Surface outlined model of AlphaFold2 predicted model of the LapB-LpxC complex with LapB homodimer.Magnified structural dissection depicting potential residues within LapB TPR 2-4 that may be critical for LapB-LpxC interaction (5, 6).

**Supplementary Figure 5.**

**
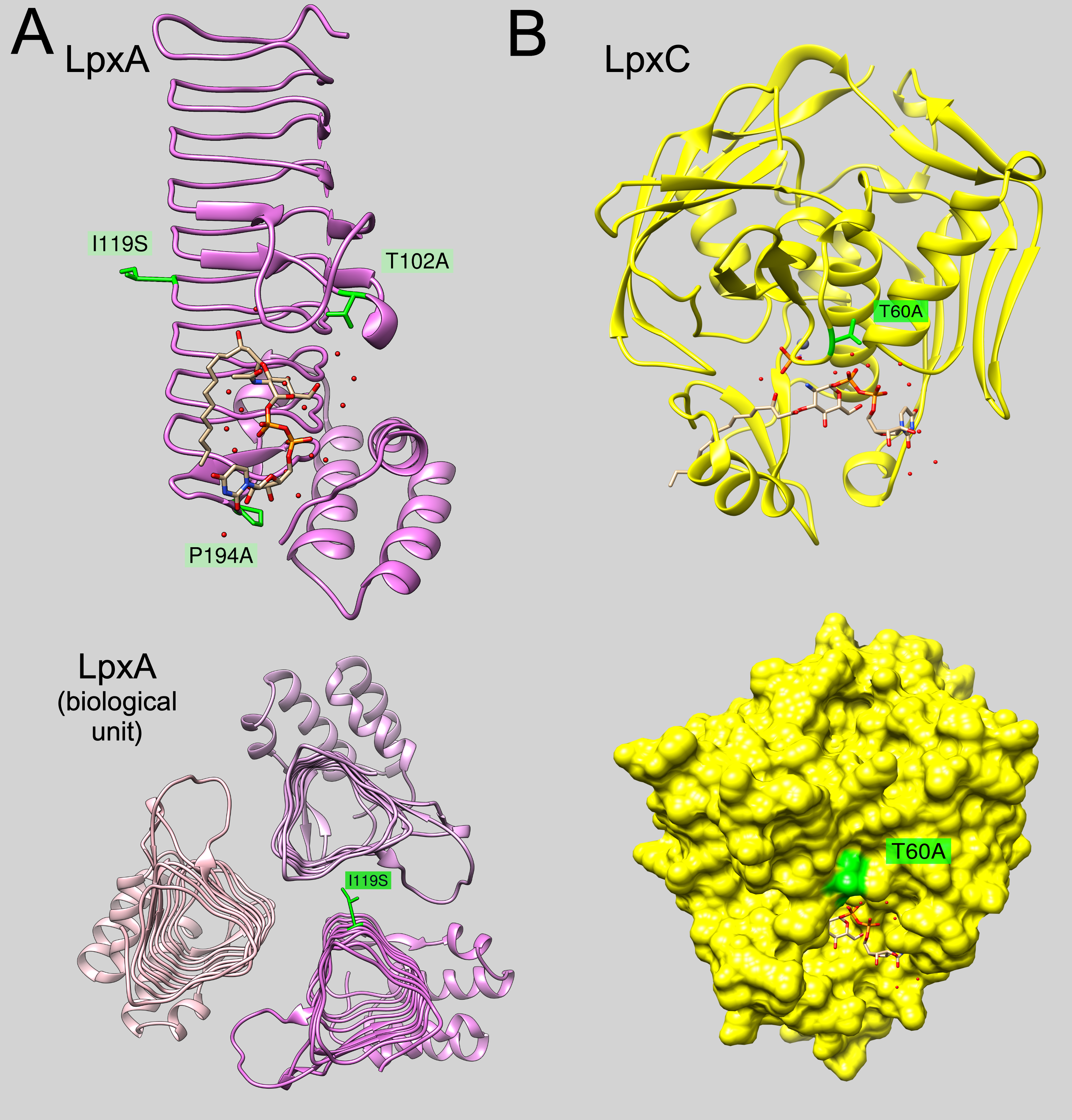
**

**Figure S5. LpxA and LpxC suppressor mutations isolated from ∆*lapB* mutants are positioned near critical interfaces of catalytic activity or structure.** (**A**) AlphaFold2 predicted *S*. Typhimurium LpxA monomer and trimer (biological unit) with identified SNP’s (green) isolated from the Δ*lapB* mutant suppressor screen in ribbon presentation for visual clarity (**Table S3**). (**B**) AlphaFold2 predicted *S*. Typhimurium LpxC (yellow) depicting the T60A SNP (green) isolated from the from the Δ*lapB* mutant suppressor screen (**Table S3**) (5, 6).

**Tables**

**Table S1.** Bacterial strains and plasmids used in this study

| Strain | Genotype and Notes | Reference |
| --- | --- | --- |
| *Escherichia coli* | | |
| DH5-alpha | For plasmid recombination and cloning | ATCC |
| BL21 (DE3) | For over-expression and affinity purification | ATCC |
| *Salmonella enterica* serovar Typhimurium 14028s | | |
| ZD004 | Wild type (*lapB*^+^) *wza-lacZ* | (14-16) |
| ZD0036 | ∆*lapB::tetRA* | This study |
| ZD0037 | *lapB^Flag-C^* | This study |
| ZD0038 | *∆lapB::tetRA lpxA^I119S^* (suppressor isolate 1) | This study |
| ZD0039 | *∆lapB::tetRa lpxC*^T60A^ *(*suppressor isolate 2) | This study |
| ZD0040 | *∆lapB::tetRA lpxA*^P194Q^*,^T102^* (suppressor isolate 3) | This study |
| **Plasmids** |  |  |
| pBAD24-Empty | Expression vector (pEMPTY) | (17) |
| pBAD24-LapB^6xHisC^ | LapB (1-389) transmembrane protein encoded with a carboxy-terminal polyhistidine tag | This study |
| pBAD24-LapB^∆TM6xHisC^ | LapB devoid of its TM segment (∆1-22 amino acids) encoded with a carboxy-terminal polyhistidine tag | This study |
| p_ara_: *lpxC*^Strep-N^ | Modified pBAD24 plasmid encoding LpxC devoid of C-terminal extension and replaced with a carboxy terminal Strep-Tag, | This study |
| pACYC1-Duet | BL21 IPTG-inducible co-expression vector | Commercial  (Novagen) |
| pACYC1-^6xHN^LapB^∆TM^ | Soluble form of LapB (23-389 amino acids) with an amino-terminal polyhistidine tag | This study |
| pACYC1-Empty_LpxC^∆293-305-S-Tag^ | LpxC devoid of C-terminal extension and replaced with a carboxy terminal S-Tag | This study |
| pACYC1-^6xHisN^LapB^∆TM-TPR1^_Empty | Amino-terminal polyhistidine LapB (98-389) devoid of TM-TPR1 | This study |
| pACYC1-^6xHisN^LapB^∆TM-TPR1^_LpxC^∆293-305-S-Tag^ | Amino-terminal polyhistidine LapB (LapB^∆1-97^) devoid of TM-TPR1 (LapB^∆TM-TPR1^) encoded with S-tagged LpxC (LpxC^∆293-305-S-Tag^) | This study |
| pACYC1-^6xHisN^LapB^∆TM-TPR2^_Empty | Amino-terminal polyhistidine LapB devoid of TM –TPR2 (LapB^∆1-138^) | This study |
| pACYC1-^6xHisN^LapB^∆TM-TPR2^_ LpxC^∆293-305-S-Tag^ | Amino-terminal polyhistidine LapB devoid of TM –TPR2 (LapB^∆1-138^) encoded with S-tagged LpxC | This study |
| pACYC1^-6xHisN^LapB^∆TM, ∆TPR5-Stop^ | Amino-terminal polyhistidine LapB encoding only LapB TPR’s 1-4 (LapB^∆1-22, ∆214-389^) in tandem with LpxC | This study |
| pSUB11 | Used as a template for FLAG tagging into chromosomal genes with a Kanamycin resistance marker. | (18) |
| pKD46 | Arabinose-inducible pBAD24 plasmid system encoding *bet,exo,gam* genes of the lambda phage-red system for ∆*lapB::tetRA* development | (19) |

**Table S2.** Primers used in this study

| Primer Name | Primer Sequence (5’-3’) | Notes |
| --- | --- | --- |
| **pBAD24 Constructs** | | |
| LapB^6xHisC^ F1 | GCTAGCAGGAGGAATTCACCATGTTGGAGTTGTTATTTCTGTTGTTGCC | For pBAD-LapB^6xHisC^ |
| LapB^6xHisC^ R2 | GGCAACAACAGAAATAACAACTCCAACATGGTGAATTCCTCCTGCTAGC |  |
| LapB^6xHisC^ R1 | CTCTAGAGGATCCCCGGGTACCTAGTGATGGTGATGGTGATGCTGCCCATCAAGTCCGCG |  |
| LapB^6xHisC^ F2 | CGCGGACTTGATGGGCAGCATCACCATCACCATCACTAGGTACCCGGGGATCCTCTAGAG |  |
| LapB^+^ R1 | CTCTAGAGGATCCCCGGGTACCTACTGCCCATCAAGTCCGC | For pBAD-LapB^+^ |
| LapB^+^ F2 | GCGGACTTGATGGGCAGTAGGTACCCGGGGATCCTCTAGAG |  |
| LapB^∆TM6xHisC^ F1 | CATCACCACAGCCAGGATCCTGCGCAACAAACAAAACAGGATG | For pBAD-LapB^∆TM6xHisC^ |
| LapB^∆TM6xHisC^ R2 | CATCCTGTTTTGTTTGTTGCGCACATGGTATATCTCCTTATTAAAGTTAAACAAAATTATTTCT |  |
| LapB^∆TM6xHisC^ R1 | CTCTAGAGGATCCCCGGGTACCTAGTGATGGTGATGGTGATGCTGCCCATCAAGTCCGCG |  |
| LapB^∆TM6xHisC^ F2 | CGCGGACTTGATGGGCAGCATCACCATCACCATCACTAGGTACCCGGGGATCCTCTAGAG |  |
| pBAD-Seq_F | CTGTTTCTCCATACCGTT | PBAD24 sequencing validation |
| pBAD-Seq_R | GGCTGAAAATCTTCTCT |  |
| *lapB^FLAG-C^ F* | GGCATGGTCGACCATTAAACCTATTCGCGGACTTGATGGGCAGGACTACAAAGACCATGACGGTGATTAT | Chromosomal epitope tagging of *lapB*^+^ |
| *lapB^FLAG-C^ R* | TGTCATTAAGTATGTTGTAACTAAAGCGAGGCTTTTTTTATATACATATGAATATCCTCCTTAGTTCCTATTC |  |
| ^StrepN^LpxC F | GCTAGCAGGAGGAATTCACCATGTGGAGCCATCCGCAGTTTGAAAAAATCAAACAAAGGACACTTAAACGTATCGTT | For pBAD-^StrepN^LpxC |
| ^StrepN^LpxC R | AACGATACGTTTAAGTGTCCTTTGTTTGATTTTTTCAAACTGCGGATGGCTCCACATGGTGAATTCCTCCTGCTAGC |  |
| **pACYC1-Duet Constructs** | | |
| ^6xHisN-^LapB^∆TM^ F1 | CATCACCACAGCCAGGATCCTGCGCAACAAACAAAACAGGATG | For pACYC1-^6xHisN-^LapB^∆TM^ |
| ^6xHisN-^LapB^∆TM^ R2 | CATCCTGTTTTGTTTGTTGCGCAGGATCCTGGCTGTGGTGATG |  |
| ^6xHisN-^LapB^∆TM^ R1 | GCGCCGAGCTCGAATTCCTACTGCCCATCAAGTCCGC |  |
| ^6xHisN-^LapB^∆TM^ F2 | GCGGACTTGATGGGCAGTAGGAATTCGAGCTCGGCGC |  |
| LpxC^∆293-305-S-Tag^ F1 | CATCTTAGTATATTAGTTAAGTATAAGAAGGAGATATACATATGATCAAACAAAGGACACTTAAACGTATCGTT | For pACY1-EMPTY_ LpxC^∆293-305-S-Tag^ |
| LpxC^∆293-305-S-Tag^ R2 | AACGATACGTTTAAGTGTCCTTTGTTTGATCATATGTATATCTCCTTCTTATACTTAACTAATATACTAAGATG |  |
| LpxC^∆293-305-S-Tag^ R1 | TCGCAGCAGCGGTTTCTTTCAGTTCTGCGTCGTCCTGG |  |
| LpxC^∆293-305-S-Tag^ F2 | CCAGGACGACGCAGAACTGAAAGAAACCGCTGCTGCGA |  |
| ^6xHisN^LapB^∆TM-TPR1^ F1 | CATCACCACAGCCAGGATCCGGAAAGCGCTTCATTGACCTATGAACAG | For pACY1- ^6xHisN^LapB^∆TM-TPR1^_ LpxC^∆293-305-S-Tag^ |
| ^6xHisN^LapB^∆TM-TPR1^ R2 | CTGTTCATAGGTCAATGAAGCGCTTTCCGGATCCTGGCTGTGGTGATG |  |
| ^6xHisN^LapB^∆TM-TPR2^ R1 | CATCACCACAGCCAGGATCCGTTTCGCGTAGGCGCGTTAC | For pACY1- ^6xHisN^LapB^∆TM-TPR2^_ LpxC^∆293-305-S-Tag^ |
| ^6xHisN^LapB^∆TM-TPR2^ F1 | TAACGCGCCTACGCGAAACGGATCCTGGCTGTGGTGATG |  |
| ^6xHN^LapB^∆TM,∆TPR5-Rdx^F1 | GCGCCGAGCTCGAATTCCTAACTGCGCACCTGCTCG | For pACYC1-^6xHisN^LapB^∆^ ^∆TM,∆TPR5-Rdx^ |
| ^6xHN^LapB^∆TM,∆TPR5-Rdx^ R1 | TGCCGCCGCAGATAAAAATAGCTAGGAATTCGAGCTCGGCGC |  |

**Table S3.** SNPs in LpxA and LpxC isolated from ∆lapB mutants variably restored the growth and Rcs-activation phenotypes of the *lapB*-deleted bacteria

| *lapB* mutant suppressor isolates SNP’s | | |
| --- | --- | --- |
| Isolate # | Gene | Mutation |
| 1 | *lpxA* | Ile119Ser (356T>G) |
| 2 | *lpxA* | Thr102Ala (304A>G) |
|  | *lpxC* | Thr60Ala (178A>G) |
| 3 | *lpxA* | Pro194Gln (581C>A) |

**Table S4.** AlphaFold2 pLDDT multimer scores and pITM + pTM of *Salmonella LapB-LpxC* interactions and affinity-tagged *Salmonella* ^6xHN^LapB^∆TM^ + LpxC^∆293-305-S-Tag^

| **Protein** | **Model #** | **pLDDT^†^** | **ipTM + pTM^‡^** |
| --- | --- | --- | --- |
| *S*. Typhimurium LapB + LpxC | 1 | 88.8 | 0.88 |
|  | 2 | 88.7 | 0.88 |
|  | 3 | 87.8 | 0.88 |
|  | 4 | 88.4 | 0.88 |
|  | 5 | 88.6 | 0.89 |
| ^6xHN^LapB^∆TM^+  LpxC^∆293-305-S-Tag^ | 1 | 87.8 | 0.87 |
|  | 2 | 87.3 | 0.87 |
|  | 3 | 87.0 | 0.87 |
|  | 4 | 87.4 | 0.86 |
|  | 5 | 87.4 | 0.87 |

†: The pLDDT (predicted local distance difference test) per residue score is averaged to give the numbers shown for each respective protein.

‡: The ipTM + pTM (interface predicted template modeling + predicted template modeling) score is a measure of confidence in the predicted interaction. This is weighted 80% ipTM + 20% pTM.

**References**

1. Karp, P. D., Billington, R., Caspi, R., Fulcher, C. A., Latendresse, M., Kothari, A. *et al.* (2019) The BioCyc collection of microbial genomes and metabolic pathways Brief Bioinform **20**, 1085-1093 10.1093/bib/bbx085

2. Keseler, I. M., Gama-Castro, S., Mackie, A., Billington, R., Bonavides-Martinez, C., Caspi, R. *et al.* (2021) The EcoCyc Database in 2021 Front Microbiol **12**, 711077 10.3389/fmicb.2021.711077

3. Nicolaes, V., El Hajjaji, H., Davis, R. M., Van der Henst, C., Depuydt, M., Leverrier, P. *et al.* (2014) Insights into the function of YciM, a heat shock membrane protein required to maintain envelope integrity in Escherichia coli J Bacteriol **196**, 300-309 10.1128/JB.00921-13

4. Prince, C., andJia, Z. (2015) An Unexpected Duo: Rubredoxin Binds Nine TPR Motifs to Form LapB, an Essential Regulator of Lipopolysaccharide Synthesis Structure **23**, 1500-1506 10.1016/j.str.2015.06.011

5. Mirdita, M., Schutze, K., Moriwaki, Y., Heo, L., Ovchinnikov, S., andSteinegger, M. (2022) ColabFold: making protein folding accessible to all Nat Methods **19**, 679-682 10.1038/s41592-022-01488-1

6. Chen, L., Baker, B., Santos, E., Sheep, M., andDaftarian, D. (2019) A Visualization Tool for Cryo-EM Protein Validation with an Unsupervised Machine Learning Model in Chimera Platform Medicines (Basel) **6**, 10.3390/medicines6030086

7. Cian, M. B., Giordano, N. P., Masilamani, R., Minor, K. E., andDalebroux, Z. D. (2019) Salmonella enterica Serovar Typhimurium Uses PbgA/YejM To Regulate Lipopolysaccharide Assembly during Bacteremia Infect Immun **88**, 10.1128/IAI.00758-19

8. Klein, G., Kobylak, N., Lindner, B., Stupak, A., andRaina, S. (2014) Assembly of lipopolysaccharide in Escherichia coli requires the essential LapB heat shock protein J Biol Chem **289**, 14829-14853 10.1074/jbc.M113.539494

9. Fivenson, E. M., andBernhardt, T. G. (2020) An Essential Membrane Protein Modulates the Proteolysis of LpxC to Control Lipopolysaccharide Synthesis in Escherichia coli Mbio **11**, ARTN e00939-20

10.1128/mBio.00939-20

10. Guest, R. L., Guerra, D. S., Wissler, M., Grimm, J., andSilhavy, T. J. (2020) YejM Modulates Activity of the YciM/FtsH Protease Complex To Prevent Lethal Accumulation of Lipopolysaccharide Mbio **11**, ARTN e00598-20

10.1128/mBio.00598-20

11. Mahalakshmi, S., Sunayana, M. R., SaiSree, L., andReddy, M. (2014) yciM is an essential gene required for regulation of lipopolysaccharide synthesis in Escherichia coli Mol Microbiol **91**, 145-157 10.1111/mmi.12452

12. Nguyen, D., Kelly, K., Qiu, N., andMisra, R. (2020) YejM Controls LpxC Levels by Regulating Protease Activity o the FtsH/YciM Complex of Escherichia coli Journal of Bacteriology **202**, ARTN e00303-20

10.1128/JB.00303-20

13. Campos, P. A., Fuga, B., Ferreira, M. L., Brigido, R., Lincopan, N., Gontijo-Filho, P. P., andRibas, R. M. (2021) Genetic Alterations Associated with Polymyxin B Resistance in Nosocomial KPC-2-Producing Klebsiella pneumoniae from Brazil Microb Drug Resist **27**, 1677-1684 10.1089/mdr.2020.0531

14. Dalebroux, Z. D., Edrozo, M. B., Pfuetzner, R. A., Ressl, S., Kulasekara, B. R., Blanc, M. P., andMiller, S. I. (2015) Delivery of cardiolipins to the Salmonella outer membrane is necessary for survival within host tissues and virulence Cell Host Microbe **17**, 441-451 10.1016/j.chom.2015.03.003

15. Cian, M. B., Giordano, N. P., Masilamani, R., Minor, K. E., andDalebroux, Z. D. (2019) Salmonella enterica serovar Typhimurium use PbgA/YejM to regulate lipopolysaccharide assembly during bacteremia Infect Immun 10.1128/IAI.00758-19

16. Giordano, N. P., Mettlach, J. A., andDalebroux, Z. D. (2022) Conserved Tandem Arginines for PbgA/YejM Allow Salmonella Typhimurium To Regulate LpxC and Control Lipopolysaccharide Biogenesis during Infection Infection and Immunity **90**, ARTN e00490-21

10.1128/iai.00490-21

17. Guzman, L. M., Belin, D., Carson, M. J., andBeckwith, J. (1995) Tight regulation, modulation, and high-level expression by vectors containing the arabinose PBAD promoter Journal of bacteriology **177**, 4121-4130, <http://www.ncbi.nlm.nih.gov/pubmed/7608087>

18. Uzzau, S., Figueroa-Bossi, N., Rubino, S., andBossi, L. (2001) Epitope tagging of chromosomal genes in Salmonella Proc Natl Acad Sci U S A **98**, 15264-15269 10.1073/pnas.261348198

19. Datsenko, K. A., andWanner, B. L. (2000) One-step inactivation of chromosomal genes in Escherichia coli K-12 using PCR products Proc Natl Acad Sci U S A **97**, 6640-6645 10.1073/pnas.120163297
